# Analysis of NUDIX enzymes across fungi reveals previously unrecognized diversity

**DOI:** 10.1186/s12864-025-11778-5

**Published:** 2025-07-01

**Authors:** Zofia Pasterny, Drishtee Barua, Eugenio Mancera, Anna Muszewska

**Affiliations:** 1 Institute of Biochemistry and Biophysics, Polish Academy of Sciences, Pawinskiego 5 A, Warsaw, 02-106 Poland; 2 Department of Chemistry, Wroclaw University of Technology, Wyb. Wyspianskiego 27, Wroclaw, 50-370 Poland; 3 Doctoral School of Molecular Biology and Biological Chemistry, IBB PAS, Pawinskiego 5a, Warsaw, 02-106 Poland; 4 Departamento de Ingeniería Genética, Unidad Irapuato, Centro de Investigación y de Estudios Avanzados del Instituto Politécnico Nacional, Irapuato, Mexico

**Keywords:** NUDIX hydrolases, Fungi, Protein families, Substrate specificity, *Glomeromycota*

## Abstract

**Background:**

The NUDIX superfamily encompasses highly diverse enzymes involved in a plethora of biological functions such as mRNA metabolism, DNA repair, and lipid peroxidation. These hydrolases are found in all domains of life and show surprising versatility in terms of the substrates that they process. The knowledge about the diversity of fungal NUDIX proteins is fragmentary, being largely limited to a small number of characterized enzymes from yeasts. To address this knowledge gap systematically, we performed a detailed analysis of the NUDIX hydrolases across 183 fungal proteomes.

**Results:**

Members of six of the known NUDIX families were present in fungi being particularly abundant in Glomeromycota. Phylogenetic analysis and sequence clustering grouped fungal NUDIX enzymes in 25 subfamilies, 13 of which did not cluster with previously known enzymes. These 13 newly identified subfamilies all belong to the canonical NUDIX family, and structural comparison revealed a typical NUDIX fold with α-β-α sandwich structure. Molecular docking suggested Ap3A and Ap4A as substrates with the highest binding affinity, but their possible cellular roles remain unclear. We also found evidence of expression of most of the genes that encode these enzymes, suggesting physiological relevance.

**Conclusions:**

Our analysis offers a comprehensive perspective on the structural and sequence relationships of the NUDIX superfamily across fungi with potential to guide experimental characterization of their biological functions.

**Supplementary Information:**

The online version contains supplementary material available at 10.1186/s12864-025-11778-5.

## Background

The NUDIX superfamily of enzymes encompasses various organic pyrophosphatases capable of cleaving nucleoside diphosphates linked to any moiety into nucleoside monophosphates and organic pyrophosphates, hence their name. Initially, the superfamily was called the MutT family after its first representative, the 8-oxoGTPase central for *E. coli* DNA elimination of toxic nucleotide derivatives [1]. However, apart from their role in nucleotide pool sanitization, nowadays these enzymes are known to be involved in DNA repair processes, decapping, processing of mRNA, cell cycle regulation, cellular survival [2] and gating ion channels [3]. In agreement with the wide range of functions that they perform, the diversity of the substrates of these enzymes is high, including di- and triphosphates, nucleotide sugars, dinucleosides, diphosphoinositol polyphosphates, and RNA caps [4]. The biomedical and biotechnological potential of NUDIX proteins is starting to be recognized. Human Dcp2, NUDT2, NUDT12, and NUDT16 can be used to characterize 5′ capped RNA transcripts [1], while OsNUDX14 from *Oryza sativa* is thought to be a grain quality regulator and to determine plant development, being strongly expressed in mature leaves [5]. NUDIX enzymes with feasible druggable sites have also been identified, and therefore they could represent novel drug targets for human diseases, especially in cancer cells [2].

Despite the functional differences between these enzymes, at the structural level, all NUDIX hydrolases are characterized by an α-β-α sandwich structure with a specific NUDIX motif, which contains the catalytic site and metal-binding residues. The NUDIX motif is composed of 23 amino acids: Gx_5_Ex_7_REUxEExGU where U is a bulky amino acid (typically isoleucine, leucine, or valine), and x represents any amino acid [2, 6]. In addition, the N-terminus of the helix fold has glutamates necessary for binding divalent cations that link the NUDIX hydrolases with the pyrophosphate of the substrate. In terms of the reaction, nucleophilic substitution by water occurs at particular phosphorus atoms within a diphosphate or polyphosphate chain [7].

NUDIX enzymes are ubiquitous in all kingdoms of life and viruses [3, 6, 8], and although these proteins are evolutionarily related, they exhibit significant sequence divergence. For this reason the superfamily has been divided into ten Pfam families. Table 1 presents the characteristics of each family. In fungi there are 29 NUDIX enzymes reported at the UniProt database that are part of five of the ten families. All of these records belong to Dikarya taxons and mostly to the model yeasts *Saccharomyces cerevisiae* and *Schizosaccharomyces pombe*. Therefore, the phylogenetic distribution and functional roles of NUDIX enzymes in fungi remain poorly understood. In this work, we identified 25 subfamilies belonging to the NUDIX superfamily throughout the fungal kingdom, 13 of which are newly identified and several appear to be only present in fungi. NUDIX enzymes are particularly abundant in the phylum Glomeromycota. The newly identified subfamilies possess characteristics typical of NUDIX hydrolases such as α-β-α sandwich structure and all belong to the canonical NUDIX family. Molecular docking suggested particular substrate preferences and mining available transcriptomic data provided evidence of their physiological relevance. Overall, our work highlights the extensive diversity of NUDIX enzymes in fungi and positions these organisms as a potential source of newly identified NUDIX enzymes for biotechnological or antifungal applications.

**Table 1:** Characteristics of the NUDIX Pfam families regarding the most common co-occurring domains, human and baker’s yeast representatives, function, and presence across kingdoms of life

Pfam	Most common coexisting domains	H. sapiens representatives	S. cervisiae representatives	Available PDB structures	Function	Presence (kingdoms)
PF00293	in majority of the cases exists as a single domain	NUDT1, NUDT2, NUDT3, NUDT4, NUDT4B, NUDT5, NUDT6, NUDT7, NUDT8, NUDT9, NUDT10, NUDT11, NUDT12, NUDT13, NUDT14, NUDT15, NUDT16, NUDT17, NUDT18, NUDT20, IDI1, IDI2	DCP2, DDP1, IDI1, NPY1, PCD1,YJR142 W, YJR142 W, YSA1	Among others: H. sapiens: NUDT1, NUDT2, NUDT7, NUDT12, NUDT15, NUDT16, IDI1 and IDI2, DDP1, DDP2, DPP3-alpha, ADP-ribose pyrophosphatase, mitochondrial 39 S ribosomal protein L46; E. coli: nudE, NADH pyrophosphatase, ADP-sugar pyrophosphatase, nudF.	various functions, but the most prominent one is sanitization of nucleotide pool	all kingdoms
PF03559	in majority of the cases exists as a single domain			EvaA 2,3-dehydratase (A. orientalis)	involved in antibiotic production pathways	mainly bacteria
PF09296	zf-NADH-PPase (PF09297), NUDIX domain (PF00293)	NUDT12, NUDT13		Peroxisomal NADH pyrophosphatase NUDT12 (H. sapiens, M. musculus)	deNADing NAD-capped RNA	eukaryotes and bacteria
PF11788	in majority of the cases exists as a single domain or along cannonical NUDIX domain (PF00293)	MRLP46	Mitochondrial ribosomal protein L46	Mitochondrial ribosomal protein L46 (H. sapiens, S. scrofa, S. cerevisiae, T. brucei, L. major, N. crassa)	role in mitochondrial protein synthesis	eukaryotes
PF13869	in majority of the cases exists as a single domain	NUDT21		Cleavage and polyadenylation specificity factor subunit 5 (H. sapiens)	3’ RNA cleavage and polyadenylation processing	eukaryotes
PF14815	HhH-GPD superfamily base excision DNA repair protein (PF14815)	MUTYH		Adenine DNA glycosylase (M. musculus, G. stearothermophilus, H. sapiens), MutT protein (B. bacteriovorus, B. fragilis, S. aureus, E. coli, B. henselae), 7,8-dihydro-8-oxoguanine-triphosphatase (K. pneumoniae)	excises inappropriately matched adenine from DNA backbone to either 8-oxoguanine or guanine	eukaryotes and bacteria
PF15916	NUDIX domain (PF00293)			MutT/nudix family protein (R. rubrum ATCC 11170)	functionally uncharacterised, takes part in thiamine biosynthetic process	eukaryotes and bacteria
PF16262	in majority of the cases exists as a single domain			Putative uncharacterized protein (J. denitrificans)	unknown	bacteria
PF16705	FERM central domain (PF00373)	Krev interaction trapped protein 1		Krev interaction trapped protein 1 (H. sapiens)	activates β1 integrin by antagonization of ICAP1 (Integrin Cytoplasmic Associated Protein-1)	eukaryotes (only animals)
PF18290	NUDIX domain (PF00293)	NUDT6 (H. sapiens)		NUDT7 (A. thaliana), NUDT6 (H. sapiens)	hydrolases catalyze the hydrolysis of nucleoside diphosphates which are often toxic metabolic intermediates and signaling molecules	eukaryotes and bacteria

## Methods

### Identification of NUDIX proteins across 183 fungal proteomes

To identify NUDIX enzymes across the fungal tree, we first searched all Pfam families in a set of 183 fungal proteomes (Supplementary file S1, sheet S1) by amino acid sequence similarity using pfam_scan.pl with default settings against Pfam database v. 36 [9]. Then, from the full set of Pfam family assignments of the 183 fungal proteomes, we extracted all proteins that matched the NUDIX families grouped in the CL0261 clan. The redundancy across the identified NUDIX sequences was first reduced with cd-hit (70% sequence identity, 90% coverage, 5 letters word length) [10]. Full protein sequences were then aligned with MAFFT v7.40 using the iterative local alignment mode [11, 12] and trimmed manually to the domain region using Jalview [13].

As a reference set of NUDIX enzymes, all human proteins with NUDIX domains were retrieved from the UniProt database (status reviewed) and all NUDIX domain-containing proteins with resolved structures were obtained from PDB [14]. The search in the PDB database was done using the accession of each of the 10 families that are annotated in Pfam as belonging to the NUDIX superfamily. The Pfam accessions were used as queries to retrieve all NUDIX enzymes in the RCSB database. This gave rise to 525 sequences. The redundant sequences were eliminated to improve the quality of clustering. The set of human and PDB sequences will be referred to as the r*eference sequence set* (see Supplementary file S1, sheet S9 to consult sequences from this set). The PDB sequences of NUDIX hydrolases include proteins from all kingdoms and viruses. Together, the reference sequence set contains representatives of all ten Pfam NUDIX families.

The identified fungal NUDIX domain sequences together with the reference sequence set and Pfam consensus sequences were then used as queries in a blastp v. 2.13.0 + search [15] against the same set of 183 proteomes using an e-value cutoff of 0.001. The blastp resulted in 7,394 fungal sequences, out of which 2,801 turned out to belong to the NUDIX superfamily. The remaining blastp hits contain domains that often coexist with the NUDIX domain (see Supplementary file S1, sheet S8 to see the list of these domains) and were identified with blastp by the full-length sequences of the reference set queries. The set of 2,801 fungal NUDIX hits together with the reference sequences was subjected to clustering based on amino acid sequence similarity using CLANS (with p-value of 1e − 10, attraction exponent value of 2 and remaining parameters set to default values). Such a clustering approach allows connecting together related sequences which are too divergent for sequence alignment and phylogenetic inference, i.e. proteins with identity levels impeding pairwise mapping with standard tools like blastp.

### Phylogenetic analysis

To trace the evolutionary relationships between NUDIX subfamilies, representative sequences from related clusters were aligned with mafft (--localpair–maxiterate 100, v7.407 [12]. The resulting alignment was then trimmed manually in Jalview and used for phylogenetic analyses. The phylogenetic tree was inferred for each of the alignments by maximum likelihood with IQTREE2 [16], considering 1000 bootstrap replicates (parameters: -B 1000) with automated model selection. The phylogenetic tree at the species level was generated with OrthoFinder [17] (parameters: mmseqs and dendroblast) for 41 species spanning the taxonomic diversity of the whole fungal kingdom. The Choanoflagellate *Monosiga brevicollis* and the fruit fly *Drosophila melanogaster* were used as outgroups. The species tree was used to represent the taxonomic distribution of individual protein families and subfamilies across the diverse fungal lineages. Trees were visualized and rendered in iToL [18].

### Sequence and structural characterization identified subfamilies

To characterize the sequence and structural properties of all fungal NUDIX subfamilies, all of the sequences were scanned against all Pfam family definitions with pfamscan.pl [19] to confirm the presence of the NUDIX domain (PF00293), and look for other coexisting domains. Where available, we used NCBI Conserved Domain Database (CDD) [20] annotations for the subfamilies, since CDD enables the identification of conserved domains across multiple protein sequences and defines a more detailed classification of subfamilies of the NUDIX proteins than the Pfam database. Complete protein sequences were then aligned with MAFFT v7.407 [12] and trimmed to the domain region using Jalview [13] to construct logos of the NUDIX signature motif with WebLogo 3 [21]. Low complexity regions were predicted with the SEG tool [22], coiled-coil regions with EMBOSS pepcoil [23], disordered protein regions with IUPred3 [24], subcellular localization with WoLF PSORT [25], and transmembrane regions were identified with TMHMM [26]. For each of the newly identified subfamilies, we also retrieved annotations of upstream and downstream genes with Entrez Direct [27] in order to verify their possible involvement in metabolic clusters.

In order to verify the taxonomic representation of the newly identified subfamilies, we extended the sequence search by using the newly identified fungal NUDIX sequences d as queries in a blastp v. 2.13.0 + search [15] against the NCBI non-redundant (NR) database with an e-value cutoff of 0.001 to collect all homologs. This set of sequences was mapped on the NCBI-Taxonomy database using the taxdump files (nodes and names) to extract their taxonomic assignment. We then calculated the percentage of fungal sequences in all of the subfamilies.

### Molecular docking for substrate identification

In order to predict the potential specificities of each of the newly identified subfamilies, molecular docking was performed. Each of the subfamilies was represented by two selected sequences and their AlphaFold2 3D structure models [28, 29]. These structures were then scanned against the FoldSeek database to find the most similar structures [30]. PyMOL was used to visualize and compare the structure of the representatives of the newly identified subfamilies with available PDB structures of NUDIX enzymes (The PyMOL Molecular Graphics System, Version 3.0 Schrödinger, LLC). Structures were trimmed to the NUDIX domain region in PyMOL and prepared for the molecular docking using pdb2pqr (parameters: --ff AMBER --ph-calc-method = propka --with-ph = xx) [31]. Molecular docking with AutoDock Vina v1.2.5 [32, 33] was performed for the twelve most common NUDIX substrates: 8-oxo-dGDP, 8-oxo-dGTP, ADP-glucose, ADP-ribose, Ap3 A, Ap4 A, Ap6 A, dATP, DHNTP, HSCoA, NAD + and NADPH. The substrates were obtained from the ChEBI database in SMILES format [28] and converted into three-dimensional PDBQT structures with Open Babel 3.1.1 [34] (parameters: *--gen3D -p xx -opdbqt*). AutoDock Vina predicts the receptor-ligand binding conformations and ranks them according to the predicted change in free energy during binding [35]. For all the proteins and ligands tested, we ensured they were at the same pH and protonation state to ensure the biological relevance of the predictions. As the NUDIX hydrolases reach their maximum activity in a mildly basic environment we consider the protomers of the tested representatives at pH 7.0, 8.0 and 9.0.

### Analysis of gene expression

Expression of the genes encoding the NUDIX proteins was verified using publicly available transcriptomic datasets of fungi. The data contained a total of 25 transcriptomes (six Mucoro- and Mortierellomycota species, four Glomero- and Basidiomycota, and five Ascomycota datasets). Description of transcriptome datasets, references and results of the gene expression analyses are available in Supplementary file S1, sheets S5, S6, S7. The data files were obtained from the ENA server in fastq format, and their quality was checked with FASTQC (v0.11.8) [36]. Adapters were trimmed using fastp (v0.19.6) with default settings [37]. The adapter-trimmed reads were aligned with reference genomes of the respective organisms (downloaded from NCBI Datasets) using Hisat2 (v2.1.0) [38]. The SAM alignment files were compressed into binary format (BAM) using samtools (v.10) [39]. The aligned reads were mapped with GFF files using StringTie (v2.1.3b) [40] to generate read abundance in the form of Transcripts per Million (TPM) values. The average TPM was calculated for each dataset and values < 1 were regarded as statistically not significant and removed. The remaining TPM values were then subjected to Z-score normalization. Finally, NUDIX enzymes from the identified families and subfamilies were searched in the expression dataset of protein-coding genes. A schematic representation of the workflow is shown in Supplementary Figure S1.

## Results

### NUDIX enzymes are widespread across the fungal kingdom

**Fig. 1:**
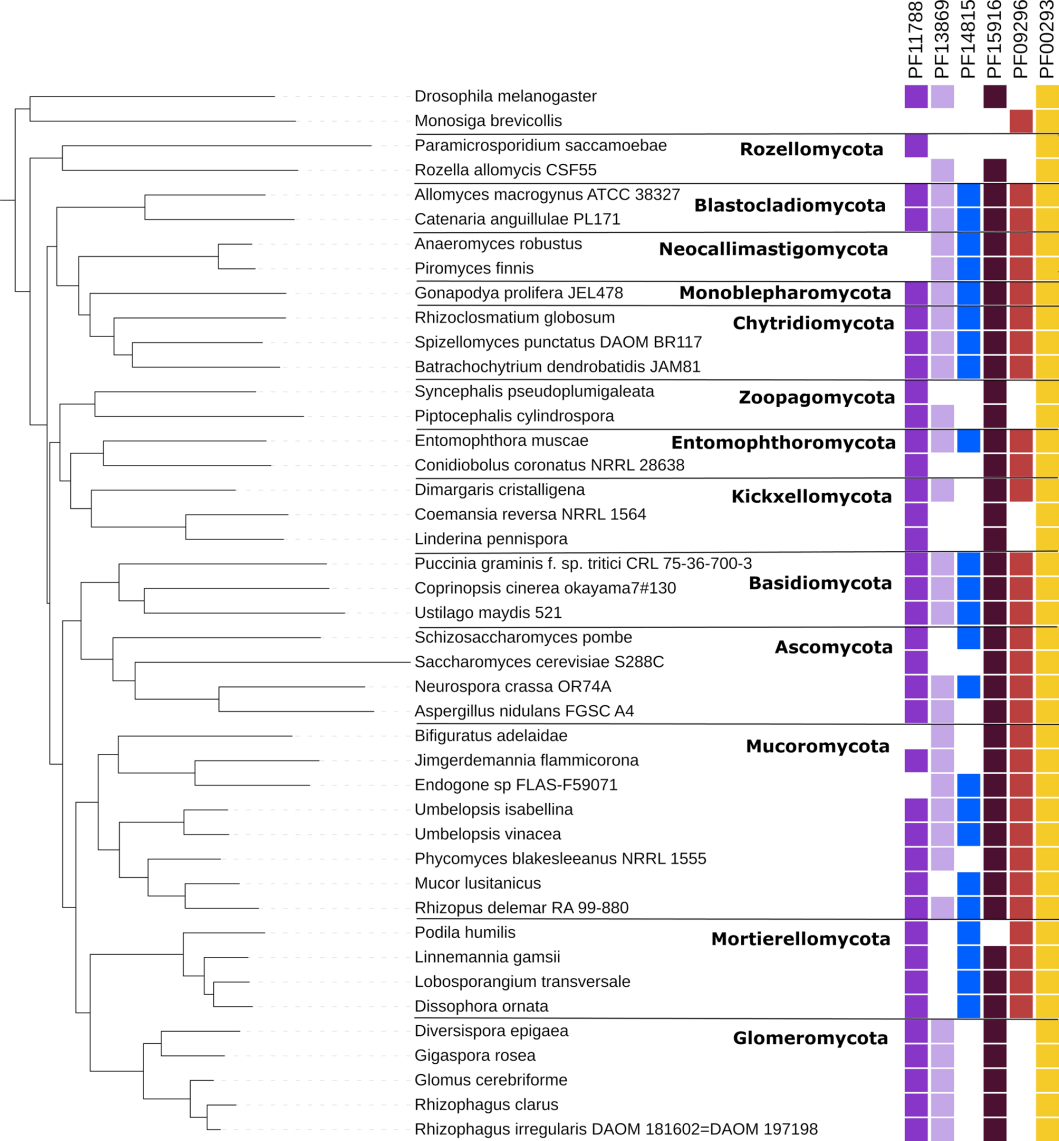
Distribution of the six NUDIX families present in fungi across phyla. The phylogenomic tree of representative fungal isolates spanning the diversity of fungi calculated with Orthofinder was annotated with the distribution of six NUDIX Pfam families. The tree is rooted with the fruit fly *D. melanogaster* and the choanoflagellate *M. brevicollis*. The figure was prepared in iToL

**Fig. 2:**
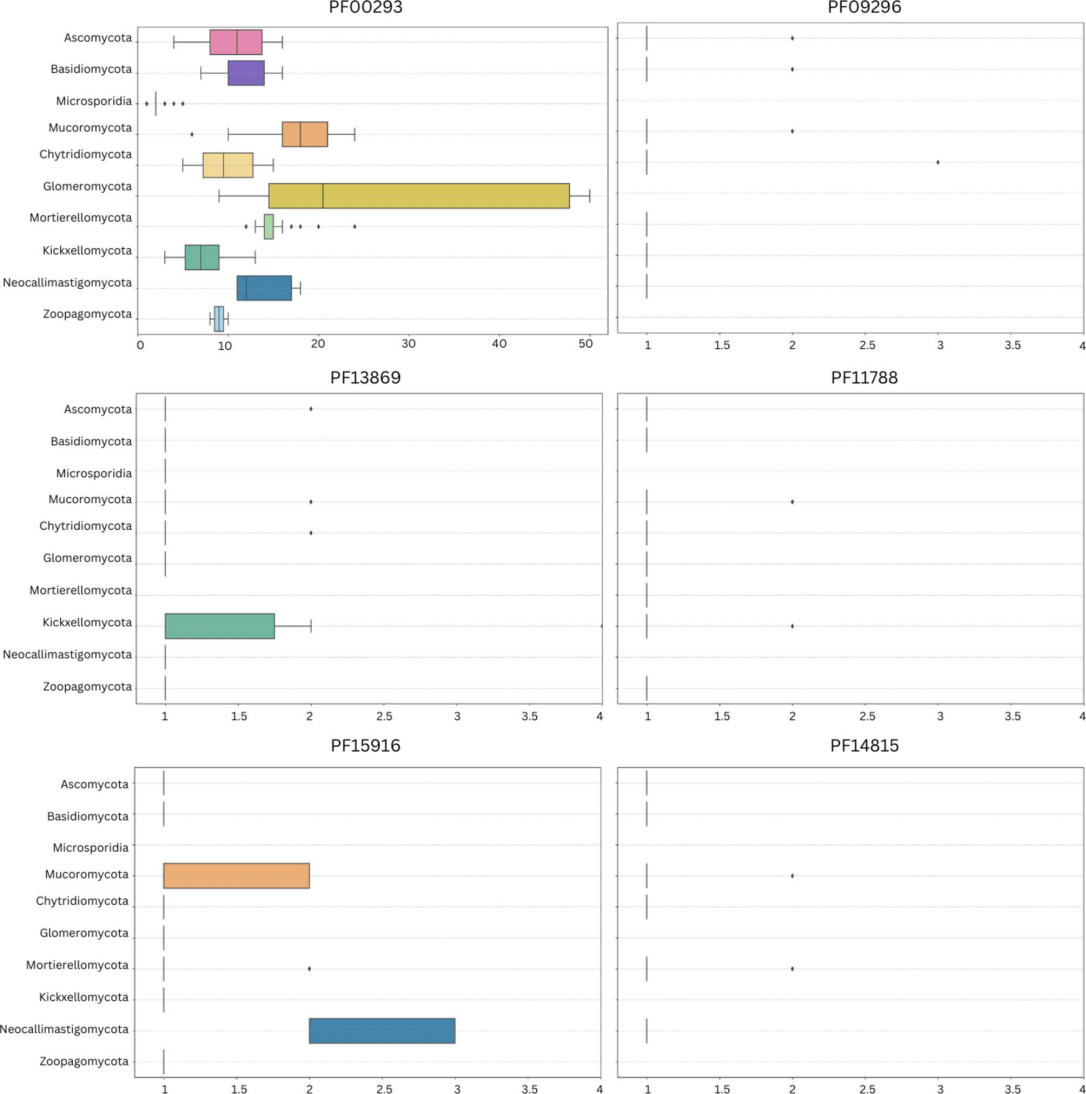
Distribution of the six NUDIX families present in fungi across phyla for 183 proteomes (listed in Supplementary file S1, sheet S1). Box plots presenting the abundance of NUDIX families across fungal phyla are shown for phyla with more than 2 representatives. In the case of proteins from the canonical NUDIX family (PF00293), the scale of the boxplot goes up to 50, leaving out the Glomeromycota representative *Gigaspora rosea*, which has 101 NUDIX enzymes. All remaining boxplots are scaled between 0 and 4 which fits all the occurrences. The central box represents the interquartile range (IQR), which contains the middle 50% of the data, spanning from the first quartile (Q1) to the third quartile (Q3). A vertical line inside the box indicates the median (Q2). Lines called whiskers extend from the box to the smallest and largest data points within 1.5 times the IQR from Q1 and Q3. Any data points beyond this range are considered outliers and are shown as individual dots

To identify NUDIX enzymes across fungi, we scanned a set of 183 fungal proteomes from all the fungal phyla for the presence of representatives of the ten NUDIX families included in the Pfam database. Six out of the ten families had homologs in the analyzed proteomes (Fig. 1), and two of the four missing families (PF03559 and PF16262) have also not been found in other eukaryotes. The canonical NUDIX domain family (PF00293) was by far the most common family present in 182 out of the 183 proteomes. This family was the only one identified in all phyla with a median number of proteins per proteome ranging from 2 for parasitic Microsporidia to more than 14 in Basidiomycota and Mucoromycota. The presence of the remaining families was less uniform (Fig. 2): the DUF4743 family (PF15916) was present in 132 proteomes, the 39 S mitochondrial ribosomal protein L46 family (PF11788) in 119, the NADH pyrophosphatase-like rudimentary family (PF09296) in 113, the NUDIX_4 domain family (PF14815) in 85, and the NUDIX_2 domain family (PF13869) in 84 proteomes. These five families had a median of close to one representative per phylum and some families were completely absent in several phyla.

Overall, the phyla Mucoromycota, Glomeromycota, and Mortierellomycota contain the highest number of NUDIX enzymes, in particular from the canonical family (PF00293). The highest abundance was observed in species from Glomeromycota, including species such as *Gigaspora rosea* (101 NUDIX proteins), *Gigaspora margarita* (47 NUDIX proteins), and *Diversispora epigaea* (50 NUDIX proteins). The phyla Ascomycota, Basidiomycota, Chytridiomycota, Mortierellomycota, and Mucoromycota have representatives of all six families present in fungi. Glomeromycota and Zoopagomycota do not have members of the families PF14815 and PF09296, Kickxellomycota lacks PF14815 NUDIX hydrolases, Microsporidia has only representatives of PF00293 and PF13869 NUDIX proteins and Neocallimastigomycota are missing proteins of the PF11788 family. Our results show that NUDIX enzymes are widespread among fungi although not all known families are present in these organisms.

### Fungal NUDIX homologs can be clustered into 25 subfamilies

To better understand the diversity of fungal NUDIX enzymes, we clustered them according to sequence similarity together with a well-curated reference set that includes representative enzymes from all kingdoms and from all the ten NUDIX Pfam families (see Methods for details). The approach allows establishing relatedness between proteins even if their sequences are too divergent to align them and perform standard phylogenetic analysis. This is particularly important for NUDIX enzymes that often have sequence similarity below 35% even though they belong to the same family or superfamily. Clustering distinguished 25 groups of fungal proteins with the NUDIX domain. On the other hand, NUDIX reference sequences from four NUDIX families (PF03559, PF16262, PF16705, and PF18290) did not cluster with any fungal protein. This is in agreement with our initial finding that only six NUDIX families are present among fungi.

**Fig. 3:**
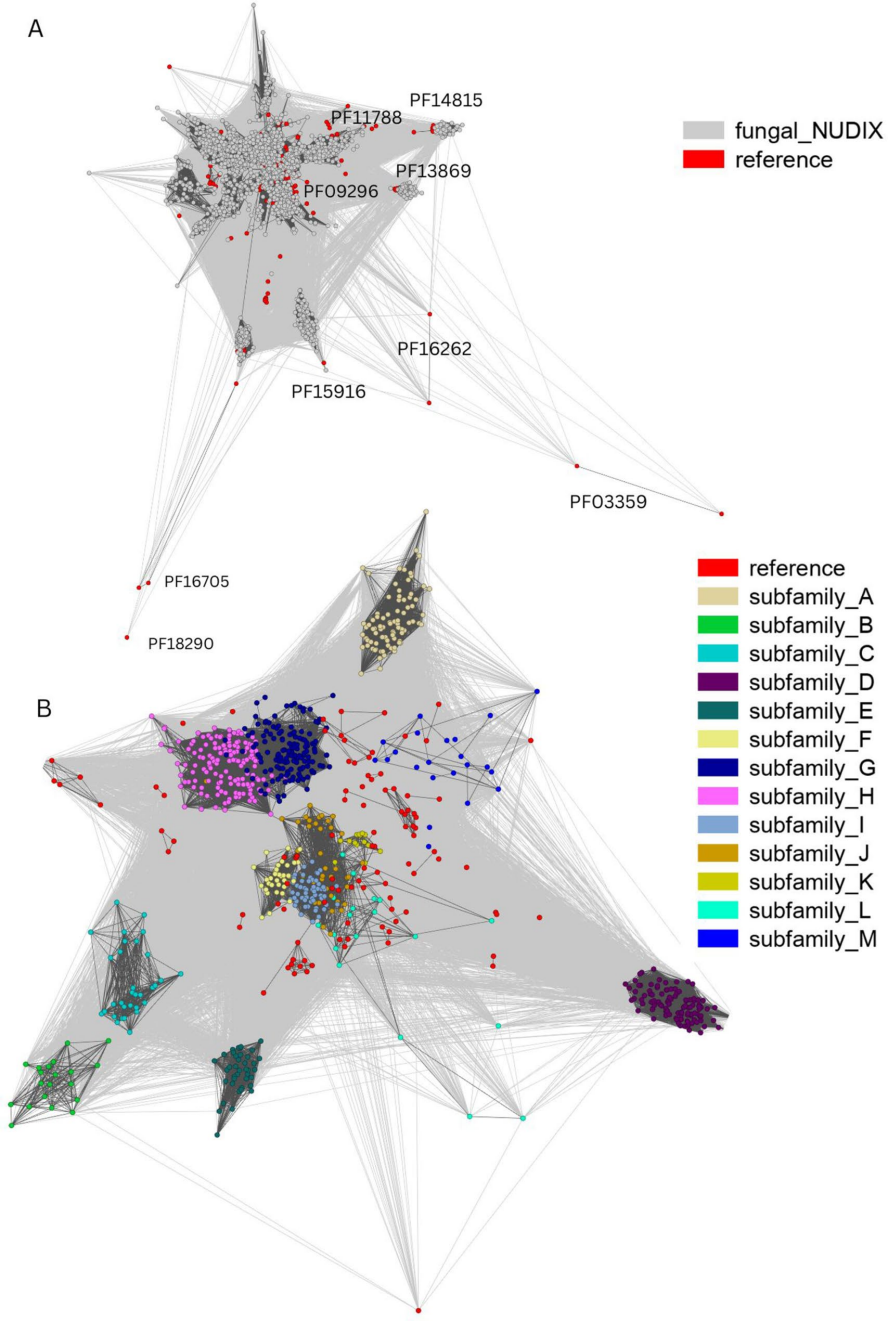
Clustering of fungal NUDIX sequences together with the reference set (human NUDIX sequences and PDB structures from all kingdoms representing the diversity of 10 Pfam NUDIX families). **A** The set of 2,801 fungal sequences identified as NUDIX hydrolases clustered by sequence similarity using CLANS. The fungal NUDIX proteins are depicted as grey nodes, reference sequences (human NUDIX proteins and PDB representatives from the ten NUDIX Pfam families) are highlighted in red. Reference sequences are marked with respective Pfam names on the Figure beside the canonical NUDIX family (PF00293) which represents the remaining enzymes. **B** Closeup of the clustering of proteins from the PF00293 where the newly identified subfamilies are marked by different colors as detailed in the figure legend

The most abundant NUDIX family in fungi, the canonical NUDIX family (PF00293) formed a large centrally located cluster (Fig. 3). In this cluster, we found homologs of nine human NUDIX proteins (IDI1 and IDI2, NUDT1, NUDT3, NUDT5, NUDT7 and NUDT8, NUDT15 and NUDT20), while ten human NUDIX hydrolases did not cluster close to any fungal homolog (NUDT4, NUDT4B, NUDT6, NUDT9, NUDT10, NUDT11, NUDT14, NUDT16, NUDT17, NUDT18, KRIT1). The latter human proteins likely have no fungal orthologs. Fungal homologs of the other five NUDIX families formed small clusters with the corresponding representatives from the reference set. Supplementary Figure S2 presents the phylogenomic tree of representative fungal isolates (spanning the fungal tree of life) annotated with the distribution of NUDIX homologs of characterized NUDIX proteins from the reference sequence set.

The clustering results were further assessed by generating maximum likelihood phylogenetic trees with representative sequences from selected clusters. Sequence diversity in the whole PF00293 family does not allow for reliable multiple sequence alignments and is of the magnitude typical for protein superfamilies with sequence identity below blastp mapping capabilities. In order to overcome this difficulty we built trees for sets of related clusters within the family sharing sequence identity in the range of 30%. The phylogenetic trees also allowed drawing hypotheses about the evolution of specific NUDIX enzymes. For example, we found that four human NUDIX hydrolases—NUDT4, NUDT4B, NUDT10, and NUDT11—are paralogs of NUDT3. In general, these findings show that there is considerable diversity of NUDIX enzymes in fungi, with representatives of 25 different subfamilies.

### 13 newly identified subfamilies of NUDIX hydrolases

Thirteen fungal NUDIX clusters formed by members of the canonical NUDIX domain family (PF00293) did not contain human or PDB representatives and, therefore, we consider them as newly identified subfamilies. We also confirmed that these newly identified subfamilies do not have Opisthokonta homologs by searching the NR database. Table 2 summarizes the characteristics of these subfamilies, Supplementary file S1, sheet S2 provides a full list of proteins associated with each subfamily, a detailed description of each of the newly identified subfamilies can be found in Supplementary file S2, and their distribution in fungal phyla is summarized in Supplementary Figure S3.

**Table 2:** Characteristics of newly identified fungal NUDIX subfamilies. From left to right, the columns correspond to the assigned subfamily name, the number of proteins in the subfamily, percentage of proteins within the subfamily with low complexity regions (LCRs), percentage of proteins with coiled coil regions, average number of amino acid residues predicted as being disordered (referred as to % residues disordered), fraction of proteins with predicted transmembrane helices (the sequences had, if any, a single transmembrane helix), the taxa in which the subfamily is present and protein domain presence beyond the NUDIX PF00293 domain (protein domain architecture is described as the number of proteins in a given subfamily having a given additional Pfam protein domain). All the values correspond to the NUDIX blastp search across 183 fungal proteomes

Subfamily	# proteins	%LCRs	% coiled coil regions	% residuesdisordered	% transmembrane helices	Taxonomic distribution	Domain architecture (Pfam names, Pfam accession)
A	84	98%	43%	51%	0%	Fungi (100%)	most have W2 (PF02020) and zf-CCCH (PF00642)
B	20	85%	10%	8%	0%	Fungi (84%), archaea (10%), and bacteria (6%)	most have IF-2B (PF01008) domain
C	32	69%	0%	5%	9%	Fungi (100%)	19 have dNK (PF01712) domain
D	105	70%	18%	9%	2%	Fungi (84%), Protozoa (13%), plants (3%)	four have diverse additional domains
E	36	89%	8%	23%	0%	Fungi (100%)	two have diverse additional domains
F	36	67%	6%	13%	0%	Fungi (100%)	19 have dNK (PF01712) domain, four have KAP_NTPase (PF07693) domain
G	140	47%	0%	13%	3%	Bacteria (48.72%), Fungi (45.80%), Insects	eight have diverse additional domains
H	152	73%	0%	14%	39%	Bacteria (49%), Fungi (46%), Insects (5%)	seven have diverse additional domains
I	57	61%	25%	10%	0%	Fungi (100%)	N/A
J	40	38%	10%	12%	0%	Fungi (100%)	N/A
K	14	86%	57%	18%	0%	Fungi (75%), Bacteria (25%0	nine have diverse retrotransposon related domains
L	19	79%	37%	9%	5%	Fungi (100%)	two have diverse additional domains
M	22	50%	14%	14%	0%	Fungi (97%), Bacteria (3%)	N/A

The enzymes of the newly identified subfamilies showed considerable diversity in terms of their sequence and structure (Table 2 and Supplementary file S2). For example, 40% of the members from subfamily H are predicted to have regions embedded in the cell membrane, 9/14 of subfamily K proteins have a retrotransposon related domain and most of the subfamily A proteins have both W2 (PF02020) and zf-CCCH (PF00642) domains. In terms of cellular localization, most of the members of the newly identified subfamilies are predicted to be localized in the nucleus.

**Fig. 4:**
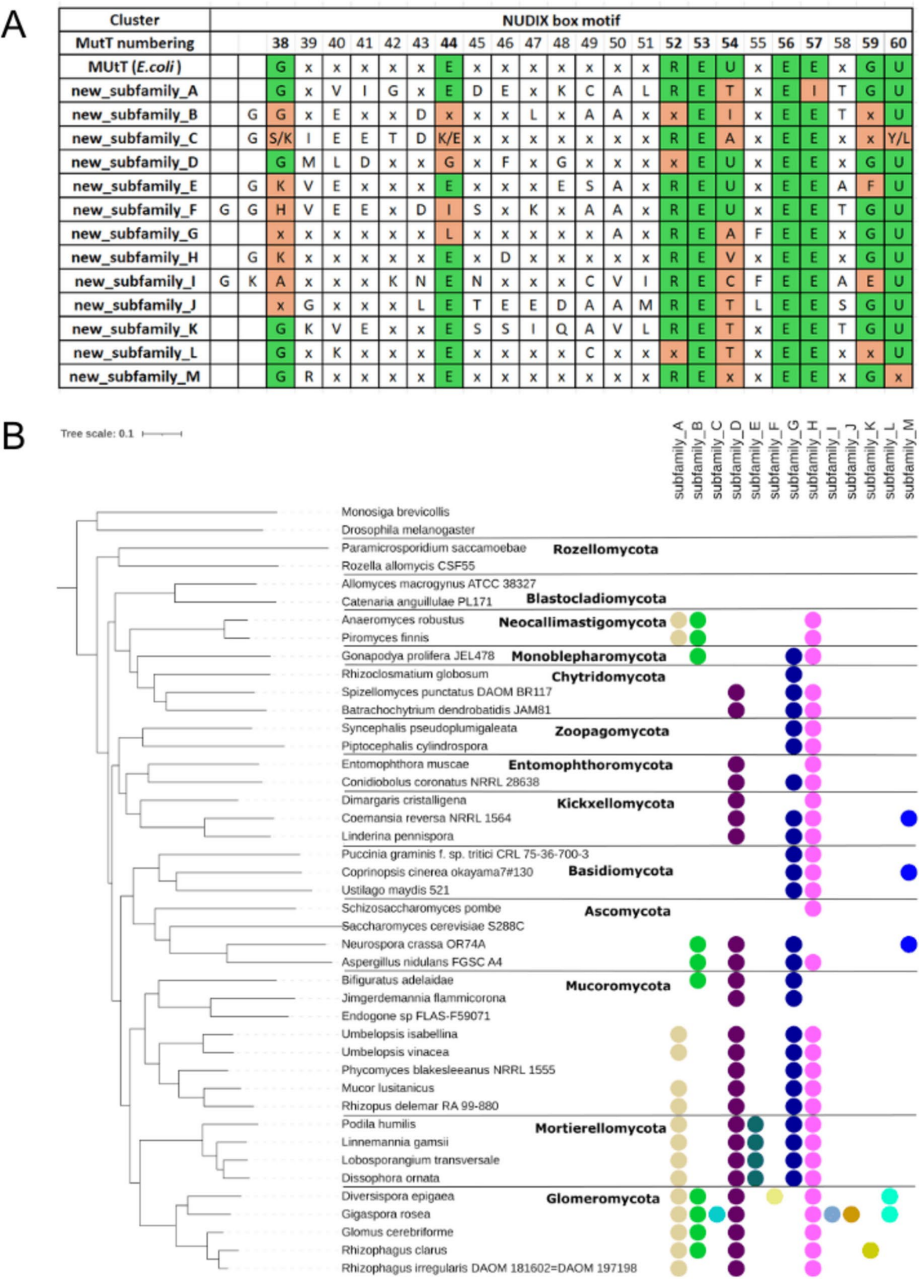
Proteins of the newly identified subfamilies within the canonical NUDIX family PF00293 show sequence diversity in the NUDIX domain and are widely distributed in the fungal kingdom. **A** Comparison of the NUDIX box motif between newly identified subfamilies, the position numbering is based on MutT protein from *E. coli* (UniProt: P08337) as reference. The cells in green indicate compatibility with the motif characteristic for the NUDIX superfamily, while the ones in orange show differences. The cells in white display the variable part of the motif. ‘U’ stands for bulky amino acid, while ‘x’ stands for any amino acid. **B** Distribution of NUDIX sequences of newly identified subfamilies in the phylogenomic tree of fungi. The tree is the same as in Fig. 1. Colored dots indicate the presence of proteins from a given subfamily in the genome of representative fungal species

All newly identified subfamilies have a well-preserved NUDIX signature motif with at least two glutamates acting as metal ligands and lysine and arginine residues that strengthen catalytic forces [7] (Fig. 4A). However, we did observe several substitutions within the NUDIX motif of the newly identified subfamilies, explaining in part why they clustered apart from previously known NUDIX enzymes. High sequence diversity within the NUDIX box is not unprecedented. This is well known for members of the PF13869 family and even the loss of the NUDIX box has been documented for other NUDIX representatives such as members of the PF14815 family [3] (Supplementary Figure S4).

**Fig. 5:**
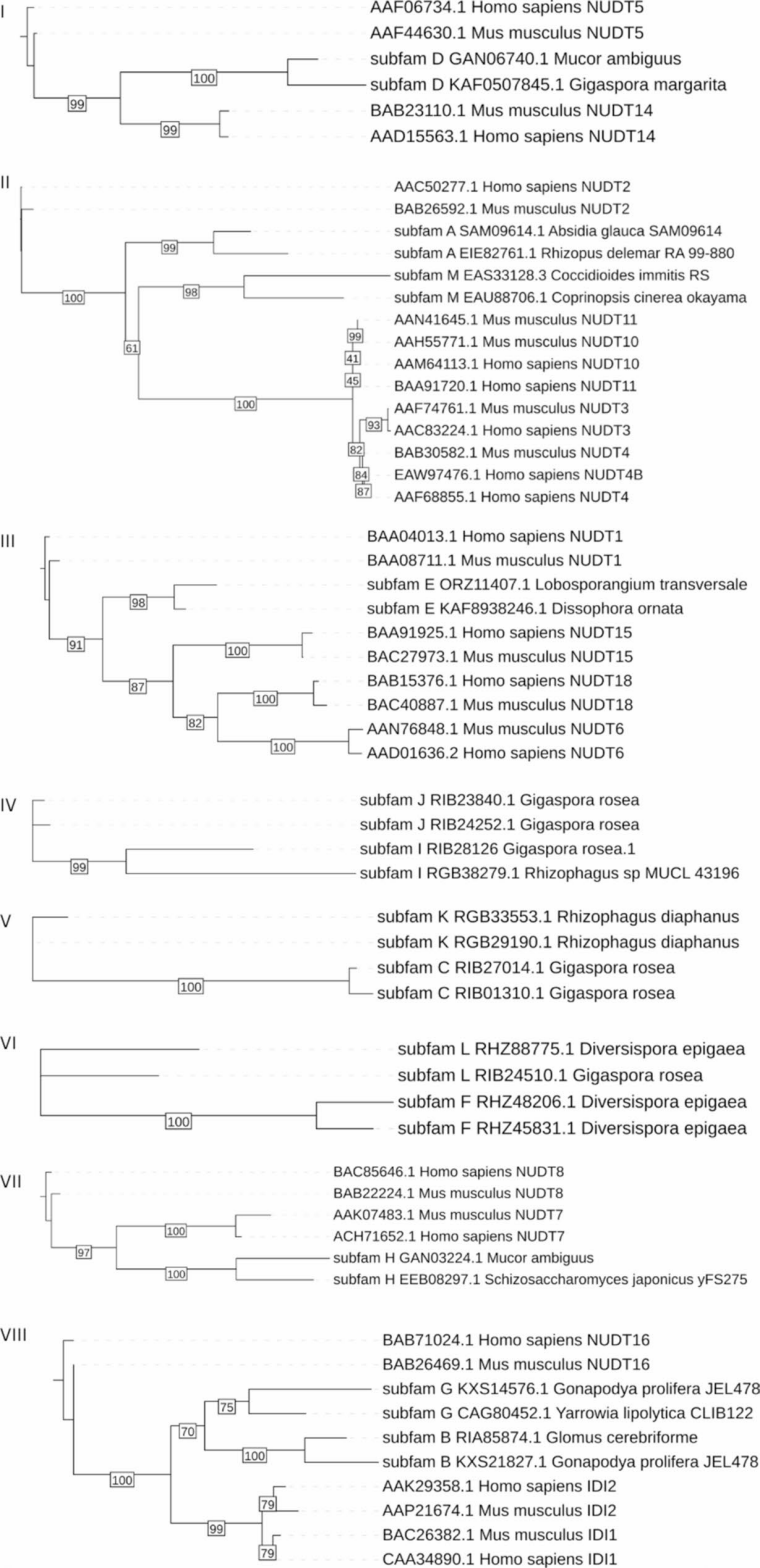
Maximum likelihood phylogenetic trees of selected sequences representing the novel fungal NUDIX clusters along with the NUDIX hydrolases from *H. sapiens* (tip labels with prefix ‘human’), and *M. musculus* (tip labels with suffix mouse). Some of the fungal subfamilies are closely related and in such cases they are on the same tree. Fungal sequences are named with the capital letter of the NUDIX subfamily they belong to, followed by the NCBI protein accession and the species name. Bootstrap values for branches are showcased in rectangular boxes

Figure 4B shows the distribution of the newly identified subfamilies across a set of genomes representing all the phyla of the fungal kingdom. Beside Rozellomycota and Blastocladiomycota, all fungal phyla have representatives of several of the newly identified subfamilies. Glomeromycota are particularly enriched with these proteins, having representatives of 11 out of 13 subfamilies. Moreover, proteins of the newly identified subfamilies are rare outside fungi, with seven of the subfamilies (A, C, E, F, I, J, L) being specific to fungi. Furthermore, the remaining subfamilies that also have representatives in other kingdoms have less than 10% of the total number of proteins from taxa that are not fungi. The former with the exception of subfamily G, for which half of the sequences in the subfamily belong to Terrabacteria representatives. Several subfamilies have very narrow distribution across the fungal kingdom. Subfamilies C, F, I, J, K, and J have representatives only in the subphylum Diversiporales, and subfamilies D and E are found only in Mortierellomycota. Even subfamilies with such narrow taxonomic distribution show sequence diversity (Supplementary Figure S5, Supplementary Figure S6). In addition, proteins from subfamilies A and M are most likely distantly related to human NUDT2 (Fig. 5A, Supplementary Figure S7, Supplementary Figure S8), which is known to regulate ApNA levels associated with stress signaling pathways and protect against oxidative damage to nucleotides.

Overall, the clustering with CLANS, the phylogenetic position, and differences in the NUDIX box motif, all support the separation of the newly identified subfamilies. Even the closest newly identified subfamilies J and K, which cluster together in CLANS, form sister clades in the phylogenetic tree and display differences in the NUDIX box motif despite their high overall sequence similarity (32.56%).

### Proteins of the newly identified fungal NUDIX subfamilies share binding affinity towards specific substrates

**Fig. 6:**
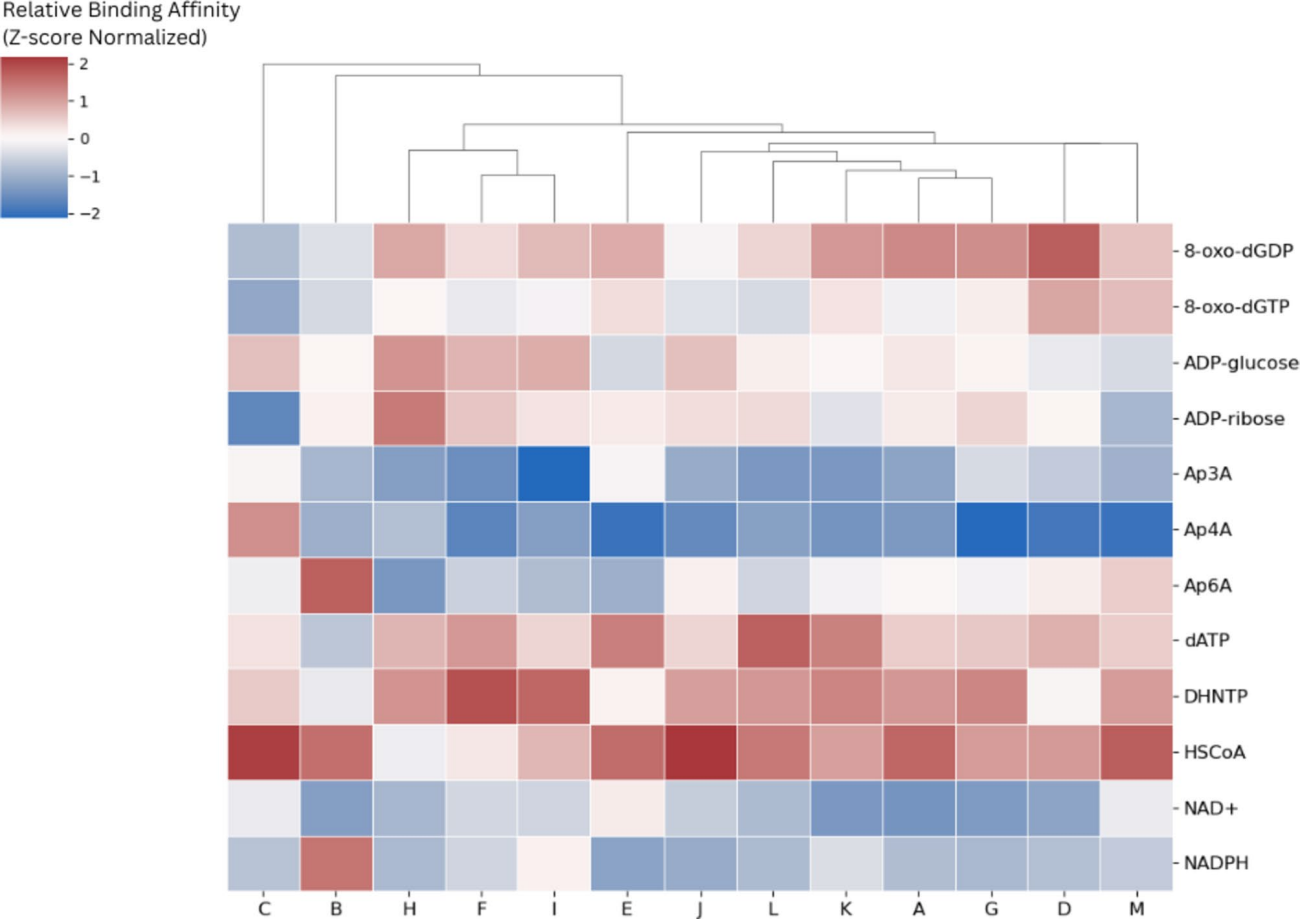
Molecular dockings show similarities in the binding affinities of the newly identified NUDIX fungal subfamilies. Molecular docking results for newly identified subfamilies against 12 substrates clustered by similarity between subfamilies. Results represent relative binding energy [kcal/mol] and are normalized by Z-scores with lower values indicating more favorable interactions as previously described [42]

NUDIX hydrolases are known to have a broad spectrum of substrates. To investigate the substrate preferences of the newly identified subfamilies, we performed molecular docking with representatives of each subfamily. The average affinity per subfamily for twelve different substrates is shown in Fig. 6 and Supplementary Figure S9. As a reference, Supplementary Figure S10 shows the average binding affinity of *H. sapiens* NUDT1 for the same substrates. Through all subfamilies, the highest binding affinity was observed towards NADPH, NAD+, Ap3 A, and Ap4 A. Despite that DHNTPase residues are conserved within the NUDIX box in the proteins from subfamily B (Supplementary Figure S11), even these proteins show preference for Ap3 A, and Ap4. These substrates are typical of human NUDT12 and NUDT13 which belong to the NUDIX family of NADH pyrophosphatase-like rudimentary NUDIX domain (PF09296). However, all sequences from the 13 newly identified subfamilies are members of the canonical NUDIX family (PF00293). Moreover, none of them possess the architecture nor the SQPWPFPXS motif that are typical of NADH pyrophosphatases and diphosphatases of the PF09296 family [41]. Therefore, it is possible that the affinity of the newly identified subfamilies has converged in parallel towards the same substrates. Subfamily C also draws attention since, unlike the rest of the subfamilies, its members show relatively low affinity to Ap4 A and high affinity to ADP-ribose [3]. The latter specificity is known in proteins involved in post-translational modification removal (NUDT16) and DNA repair (NUDT5). Even when empirical validation is needed to determine the exact function of these newly identified subfamilies, our docking results allow formulating predictions that could guide experimentation.

### Most of the newly identified NUDIX proteins are expressed

To start understanding the functional role of the predicted NUDIX proteins including those from the newly identified subfamilies, we searched for evidence of expression of the genes that encode them in available transcriptomic data. Genome-wide gene expression data is widely available for Dikarya, but it is much more scarce for other fungi. Therefore, we only considered nine representative datasets for Dikarya taxa where the predicted NUDIX enzymes are present, while we included all available datasets in the NCBI SRA database for other fungi. In total, these represented transcriptomes of 25 species (Supplementary file S1, sheets S7 & S8). In these datasets, a gene was considered expressed if it had an average TPM value ≥ 1 (Methods).

**Fig. 7:**
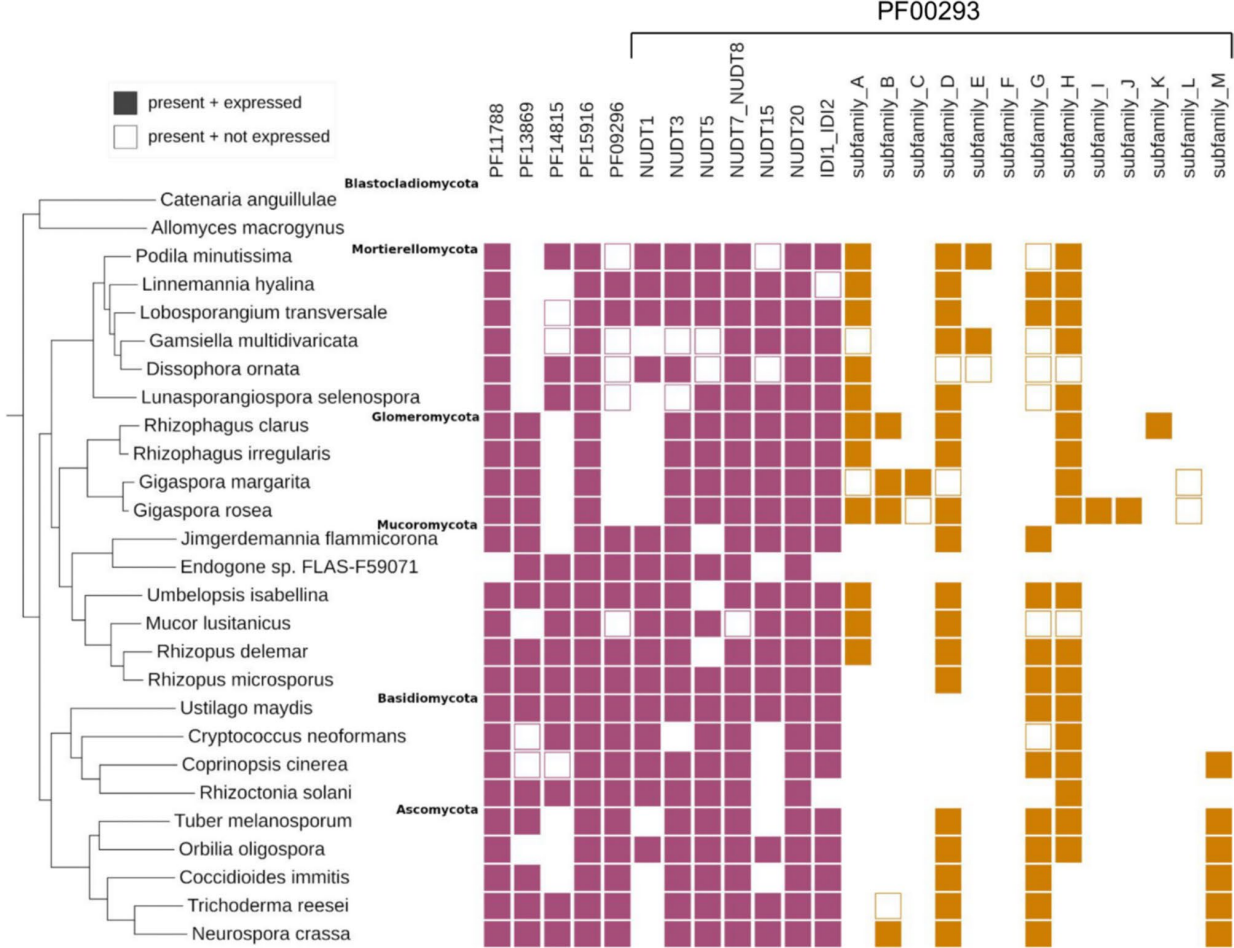
Predicted NUDIX enzymes, including those from newly identified subfamilies, are expressed across fungi. Evidence of gene expression for NUDIX families and the newly identified subfamilies in available transcriptomic datasets. Squares are only shown if the family/subfamily is present in the given taxa and they are filled if there is evidence of expression. Subfamily F is not present in the available transcriptomes

Overall, we found evidence of gene expression for 82% of all the predicted NUDIX enzymes in the 25 species with available RNASeq data. The fraction of expressed NUDIX enzymes varied between families, from 97% in the PF11788 family to 81% in the PF00293 family. At the species level, transcriptomes showed expression of a variable subset of known NUDIX members, from 6 (out of 14) in *Cryptococcus neoformans* and 6 (out of 10) in *Endogone sp.* FLAS-F59701 to 104 (out of 130) in *Gigaspora rosea*. Apart from *Rhizophagus irregularis* which had only members of the canonical NUDIX family expressed, all other species had evidence of expression of at least four of the six families (Fig. 7).

For proteins from the newly identified subfamilies, overall, we found evidence of expression for 71% of them. The former despite the fact that these subfamilies have a narrower taxonomic distribution and, in consequence, the available expression datasets of taxa that have them in their genomes is considerably limited. In total, we observed the expression of at least one member from each of the thirteen newly identified subfamilies, except for subfamily L. Transcriptomes of species of Glomeromycota showed evidence of expression of eight of the newly identified subfamilies (A, B, C, D, H, I, J, and K), followed by Mortierellomycota with six (A, B, D, E, G and H), Ascomycota with five (B, D, G, H and M), Mucoromycota with four (A, D, G and H), and Basidiomycota with three (G, H and M) (Fig. 7). For subfamilies I, J and K there was evidence of expression only in one single species. Moreover, the NUDIX genes analysed in the differential expression experiments on *R. delemar* and *G. rosea* were expressed across tested conditions pointing at a housekeeping function of many NUDIX hydrolases (Supplementary file S1, sheets S7 & S8). In summary, mining of available transcriptomes showed that the NUDIX enzymes that we predicted are widely expressed in fungi suggesting that they may be of biological importance for these organisms.

## Discussion

Even when NUDIX enzymes were first described more than 70 years ago and despite the many biological functions that they perform, these proteins have been barely studied in fungi, especially beyond Dikarya. In this work, we showed the richness of the NUDIX superfamily across fungi and discovered previously unidentified subfamilies unique to these organisms. There are only 29 fungal NUDIX enzymes with status reviewed at the UniProt database. These belong to five of the ten known NUDIX families (22 canonical NUDIX domain (PF00293), one NADH pyrophosphatase-like rudimentary NUDIX domain (PF09296), three 39 S mitochondrial ribosomal protein L46 (PF11788), one NUDIX_4 domain (PF14815), and two DUF4743 domain (PF15916)). Our work significantly expands the currently known catalog of NUDIX hydrolases in fungi by identifying 2,801 candidate enzymes, including those listed in UniProt as well as enzymes from an additional family (PF13869, NUDIX_2) not reported for fungi in this database. We did not find representatives of four NUDIX families in the 183 fungal genomes that were analyzed, which was consistent with the fact that there are no fungal NUDIX proteins associated with these families in Uniprot or Pfam. Furthermore, all of the reviewed records of fungal NUDIX hydrolases in UniProt belong to Ascomycota species, while we showed that these enzymes are present across all fungal phyla, with Glomeromycota possessing the highest number and diversity of these enzymes in fungi.

Performing detailed sequence similarity analyses we found that the fungal enzymes clustered in 25 subgroups, and that thirteen represent newly identified subfamilies as their members did not cluster with any previously known NUDIX enzyme. All of the newly identified subfamilies belong to the well-known canonical NUDIX family (PF00293). We found evidence that many of the genes that encode the newly identified subfamilies are expressed, suggesting physiological relevance. However, given the many functions that NUDIX enzymes perform, the specific cellular role of these enzymes is difficult to predict based on sequence properties. We observed common substrate preference for diadenosine triphosphate and tetraphosphate (Ap3 A and Ap4 A) in these enzymes. The level of both diadenosine metabolites is known to increase upon exposure to various stresses in bacteria and humans. Therefore they are considered to be alarmones, triggering a stress response that [43] influences a variety of cellular processes such as gene expression and DNA repair [43, 44] Therefore, it is possible that the enzymes of the newly identified subfamilies may modulate the response to cellular stress in fungi. Alternatively, they could also be involved in metabolic pathways for nucleotide synthesis and degradation. Ap3 A and Ap4 A signaling in animals is needed for apoptosis and development. Programmed cell death types differ among fungi [45] including apoptosis-like process, heterokaryon incompatibility, and ferroptosis which is a consequence of iron release and lipid peroxidation. The diversity of fungal developmental strategies, death types, and lipids could also depend, at least partially, on the diversification of the repertoire of NUDIX enzymes.

Some of the subfamilies showed preference for ADP-ribose which can also be related with DNA repair or signaling regulation. ADP-ribosylation is often found as a posttranslational modification of proteins [46]. On the other hand, poly-ADP-ribose can accumulate when DNA is damaged [47]. There are known ADP-ribose processing NUDIX enzymes that can remove it from proteins reversing glycosylation (NUDT16), and mono ADP-ribose can be converted to ribose-5-phosphate plus AMP/ATP (NUDT5). The newly identified subfamily C may extend this list of ADP-ribose processing NUDIX proteins.

NUDIX enzymes from the newly identified subfamilies are particularly abundant in Glomeromycota. Fungi in this phylum are known to lack several DNA repair and replication genes [48] which could be associated with the abundance of NUDIX enzymes. Alternatively, NUDIX enzymes in Glomeromycota could be involved in lipid metabolism since the phylum is also known to be unique in this respect [49] and NUDIX enzymes involved in beta-oxidation of fatty acids are widespread in fungi [50].

In terms of the evolutionary origin of fungal NUDIX enzymes, members of all of the six NUDIX families that we identified in fungi are also present in animals. On the other hand, animals have representatives of two families that we did not find in fungi (PF16705 and PF18290). Since other eukaryotic taxa do have members of the Nudix_hydro PF18290 which is a N-terminal domain preceding the canonical PF00293 domain, its absence in fungi probably represents a lineage-specific loss. On the other hand, the PF16705 ‘Nudix or N-terminal NPxY motif-rich region of KRIT’ is limited to animals and likely represents an evolutionary novelty. Recently, during Pfam 37.1 release, a novel NUDIX domain (PF22327, Nudt16-like) was introduced for U8 snoRNA-decapping enzyme-like proteins from animals which is likely a second NUDIX family limited to this kingdom. Therefore, the NUDIX repertoire of the Opisthokonta ancestor most likely consisted of enzymes from at least the seven families present in animals and fungi (six common to both kingdoms, and Nudix_hydro missing from the analyzed fungi).

At the level of subfamilies, only members of the canonical NUDIX PF00293 family formed more than one group when clustering by sequence similarity. This showed that diversification of NUDIX enzymes in fungi has mostly occurred within this family. Seven of these subfamilies included human representatives, suggesting that the divergence of these enzymes occurred before the animal-fungi split. The distribution of the thirteen newly identified subfamilies is more scattered throughout the fungal tree. Members of six of these subfamilies are found across multiple phyla, while six are exclusive to Glomeromycota and one is restricted to Mortierellomycota. Although it is difficult to draw conclusions regarding the specific set of events that gave rise to the observed subfamily distribution in the phylogeny, it is clear that a series of gene duplications followed by sequence diversification probably occurred throughout the evolution of Glomeromycota.

## Conclusions

Our findings could have implications beyond the annotation of the undescribed enzyme universe. NUDIX hydrolases are known to be involved in the pathogenicity of a variety of bacteria [51], and there have been attempts to use them as antimicrobial targets [52]. Although much less is known about fungal pathogens, NUDIX hydrolases have been implicated in the oxidative stress response of *Cryptococcus neoformans* [53]. The restricted distribution of some of the subfamilies also opens up the possibility to employ them as taxonomic markers either in diagnostics or for biodiversity assessment. For instance, sequences belonging to the newly identified subfamily E and that were found only in Mortierellomycota seem ideal for this purpose. Recently, NUDIX hydrolases received attention as orchestrators of carotenoid biosynthesis in plants [54]. Therefore, they could also be manipulated in fungi for the production of metabolites of commercial interest. Overall, the diversity of fungal NUDIX hydrolases described here sets them as ideal targets for tailored manipulation in both medical and biotechnological settings.

## Supplementary Information

Supplementary Material 1.

Supplementary Material 2.

## Data Availability

Data is provided within the supplementary information files and deposited at https://zenodo.org/records/15100536.

## Abbreviations

NUDIX: Nucleoside diphosphates linked to x
LCR: Low complexity region
CDD NCBI: Conserved Domain Database
NR: NCBI non-redundant database
ChEBI: Chemical Entities of Biological Interest
TPM: Transcripts per Million
SRA: Sequence Read Archive
GTP: Guanosine triphosphate
GDP: Guanosine diphosphate
ADP: Adenosine diphosphate
dATP: Deoxyadenosine triphosphate
Ap3A P1,P3-Bis(5'-adenosyl) triphosphate: Diadenosine triphosphate
Ap4A P1,P4-bis(5'-adenosyl) tetraphosphate: Diadenosine tetraphosphate
Ap6A, P1,P6-bis(5'-adenosyl)hexaphosphate: Diadenosine hexaphosphate
DHNTP: 7,8-Dihydroneopterin triphosphate
HSCoA: coenzyme A (not attached to an acyl group)
NAD+: Nicotinamide adenine dinucleotide
NADPH: Nicotinamide adenine dinucleotide phosphate
NAD+: Nicotinamide adenine dinucleotide
NADPH: Nicotinamide adenine dinucleotide phosphate
