# Supplementary material for: Analysis of NUDIX enzymes across fungi reveals previously unrecognized diversity
Source: BMC Genomics. 2025 Jul 1;26:606. doi: 10.1186/s12864-025-11778-5 (PMC12210691; doi:10.1186/s12864-025-11778-5)
Supplement: Supplementary file 2 — Supplementary Material 2. [file 12864_2025_11778_MOESM2_ESM.pdf]

Analysis of NUDIX enzymes across fungi reveals previously unrecognized diversity

Zofia Pasterny[1,2], Drishtee Barua[1], Eugenio Mancera[3] and Anna Muszewska*[1]

[1] Institute of Biochemistry and Biophysics, Polish Academy of Sciences, Pawinskiego 5A, 02-106 Warsaw, Poland

[2]Department of Chemistry, Wroclaw University of Technology, Wyb. Wyspianskiego 27, 50-370, Wroclaw, Poland

[3]Departamento de Ingeniería Genética, Centro de Investigación y de Estudios Avanzados del Instituto Politécnico Nacional, Unidad Irapuato, Irapuato, Mexico

Supplementary Figure S1. Workflow of the computational strategy to identify NUDIX proteins and assign them possible biological functions. 2

Supplementary Figure S2. Distribution of NUDIX subfamilies across selected organisms. 3

Supplementary Figure S3. The distribution of the NUDIX hydrolases from newly identified subfamilies over fungal phyla. 4

Supplementary Figure S4. NUDIX box motif conservation across all identified NUDIX subfamilies in fungi. 5

Supplementary Figure S5. Alignment of sequences from new_subfamily_D. 6

Supplementary Figure S6. Alignment of sequences from new_subfamily_F. 7

Supplementary Figure S7. Alignment of sequences from new_subfamily_A and human NUDT2 (PDB: 3U53). 8

Supplementary Figure S8. Molecular docking of the NUDIX domain with Ap4A. 9

Supplementary Figure S9. Heatmap clustering of binding affinities 10

Supplementary Figure S10. Heatmap clustering of binding affinities with control enzyme (NUDT1 from Homo sapiens - UniProt: P36639) at pH 8.0 11

Supplementary Figure S11. Conservation of DHNTPase residues 12

Supplementary characterization of subfamilies 14

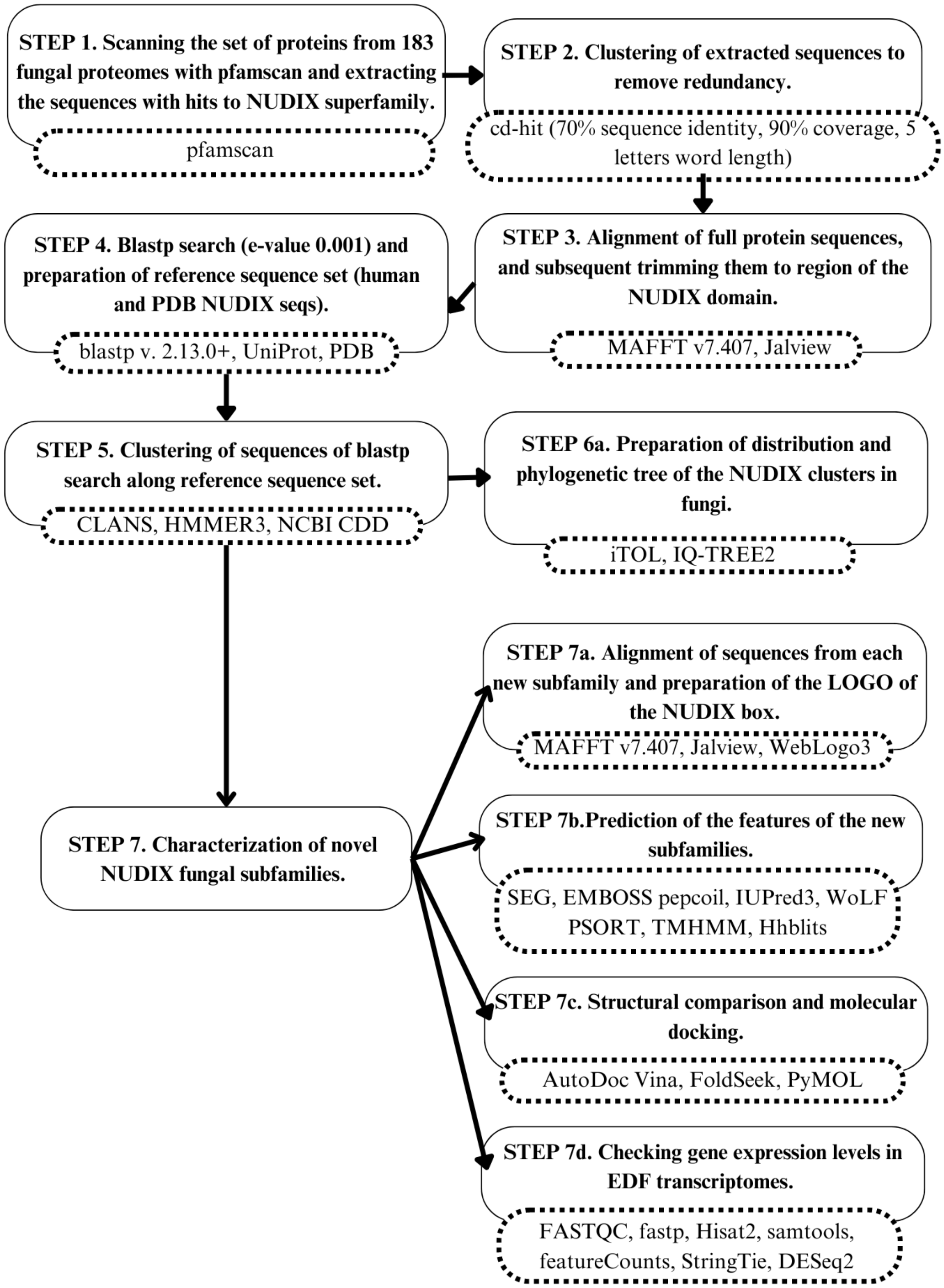

Supplementary Figure S1. Workflow of the computational strategy to identify NUDIX proteins and assign them possible biological functions.

Each box explains the analysis performed while the dotted boxes specify the computational tools employed for each step.

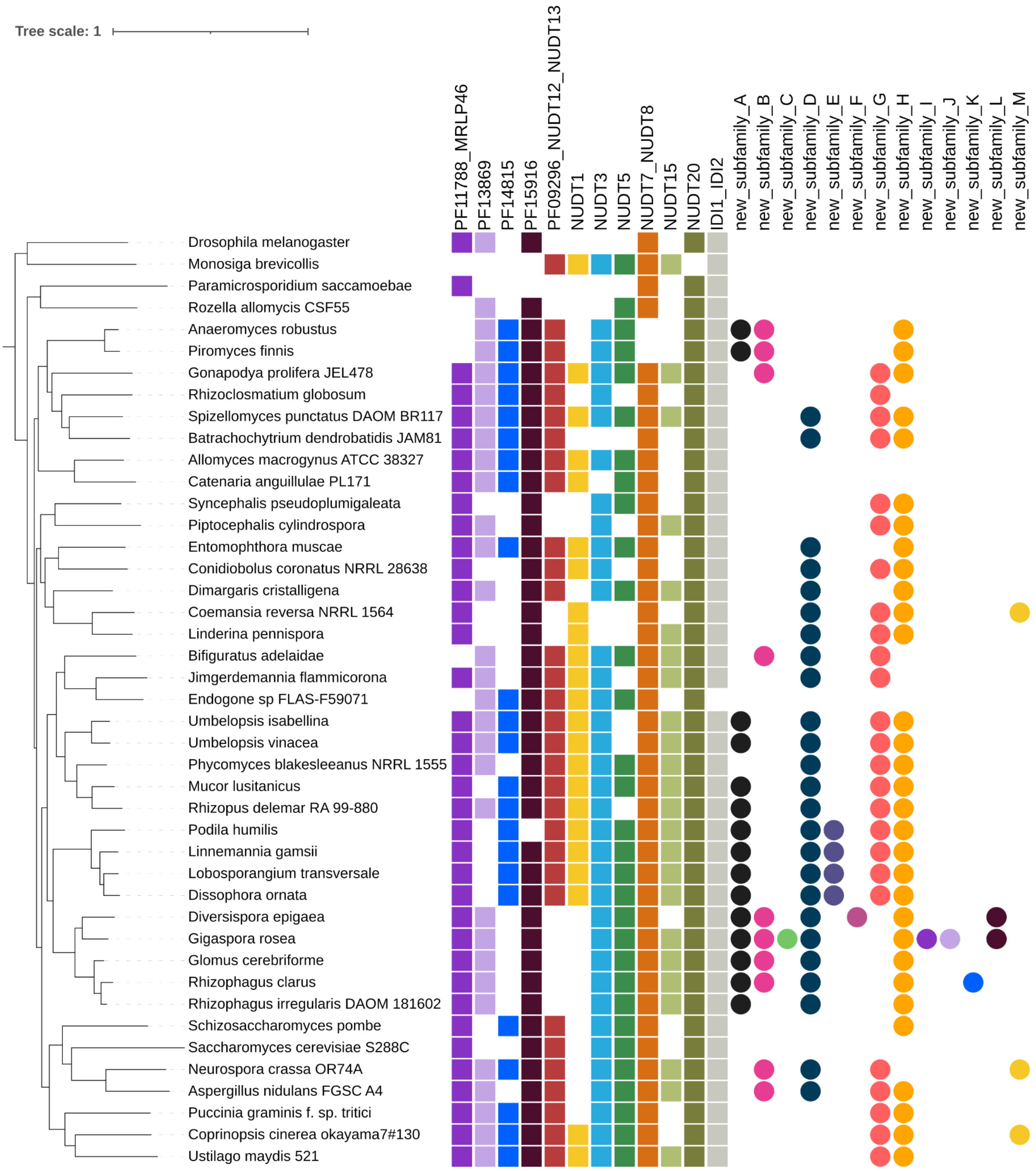

Supplementary Figure S2. Distribution of NUDIX subfamilies across selected organisms. Phylogenomics tree of representative taxa for each of the main fungal lineages computed with OrthoFinder using *Monosiga brevicollis* and *Drosophila melanogaster* as an outgroup annotated with the distribution of NUDIX families and subfamilies. NUDIX families and subfamilies with human homologs represented as squares, and newly identified subfamilies marked as circles.

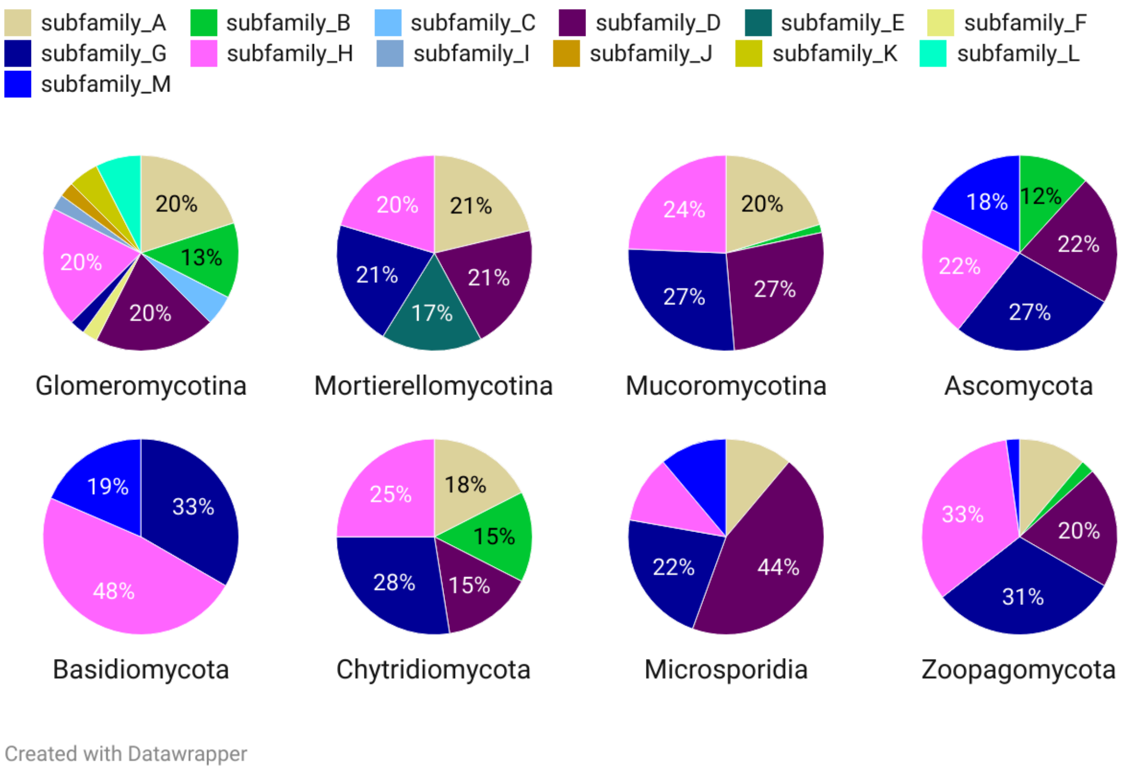

Supplementary Figure S3. The distribution of the NUDIX hydrolases from newly identified subfamilies over fungal phyla.

Phyla with a marginal number of copies were left out.

A

Cluster		NUDIX box motif																								
NUDIX motif				G	x	x	x	x	x	E	x	x	x	x	x	x	x	R	E	U	x	E	E	x	G	U
Newly identified NUDIX subfamilies	new_subfamily_A			G	x	V	I	G	x	E	D	E	x	K	C	A	L	R	E	T	x	E	I	T	G	U
	new_subfamily_B		G	S/G	x	E	x	x	D	x	x	x	L	x	R/A	A	x	x	E	I	x	E	E	T	x	U
	new_subfamily_C		G	S/K	I	E	E	T	D	K/E	x	x	x	x	x	x	x	R	E	C/A	x	E	E	x	x	Y/L
	new_subfamily_D			G	M	L	D	x	x	G	x	F	x	G	x	x	x	x	E	U	x	E	E	x	G	U
	new_subfamily_E		G	K	V	E	x	x	x	E	x	x	x	E	S	A	x	R	E	U	x	E	E	A	F	U
	new_subfamily_F	G	G	H	V	E	E	x	D	I	S	x	K	x	A	A	x	R	E	U	x	E	E	T	G	U
	new_subfamily_G			x	x	x	x	x	x	L	x	x	x	x	x	A	x	R	E	T/A	F	E	E	x	G	U
	new_subfamily_H		G	K	x	x	x	x	x	E	x	D	x	x	x	x	x	R	E	T/V	x	E	E	x	G	U
	new_subfamily_I	G	K	A	x	x	x	K	N	E	N	x	x	x	C	V	I	R	E	C	F	E	E	A	E	U
	new_subfamily_J			x	G	x	x	x	L	E	T	E	E	D	A	A	M	R	E	T	L	E	E	S	G	U
	new_subfamily_K			G	K	V	E	x	x	E	S	S	I	Q	A	V	L	R	E	T	x	E	E	T	G	U
	new_subfamily_L			G	x	K	x	x	x	E	x	x	x	x	C	x	x	x	E	T	x	E	E	x	x	U
	new_subfamily_M			G	R	x	x	x	x	E	x	x	x	x	x	x	x	R	E	x	x	E	E	x	G	x
Subfamilies associated with reference sequences	NUDT1			G	K	x	E	x	x	E	x	x	x	x	x	A	x	R	E	U	x	E	E	x	G	U
	NUDT3			G	x	x	x	x	x	E	x	x	x	x	x	A	x	R	E	x	x	E	E	A	G	U
	NUDT5		G	L	x	D	x	x	E	x	x	x	x	x	x	A	x	R	E	U	x	E	E	x	G	U
	NUDT7_NUDT8	G	G	K	x	x	x	x	D	x	x	x	x	x	T	A	x	R	E	x	x	E	E	x	x	U
	NUDT15			G	x	L	x	x	x	E	x	F	x	x	C	A	x	R	E	U	x	E	E	T	x	U
	NUDT20			G	K	x	x	x	x	E	x	x	x	x	C	A	x	R	E	U	x	E	E	x	G	U
	PF009296			G	F	x	x	x	x	E	x	x	E	x	x	x	x	R	E	U	x	E	E	x	G	U
	PF15916			G	G	I	G	F	G	H	S	P	F	E	T	V	I	K	E	C	M	E	E	A	S	I
	PF11788			x	x	x	x	x	x	E	x	L	x	x	x	x	x	R	x	U	x	E	E	x	G	x
	PF13869		G	x	x	L	x	x	x	x	D	E	x	x	x	x	x	x	x	U	x	x	x	L	x	P
	IDI1_IDI2	NUDIX box is highly divergent across different species, and it does not follow the typical NUDIX motif																								
	PF14815	does not contain conserved NUDIX box motif																								

B

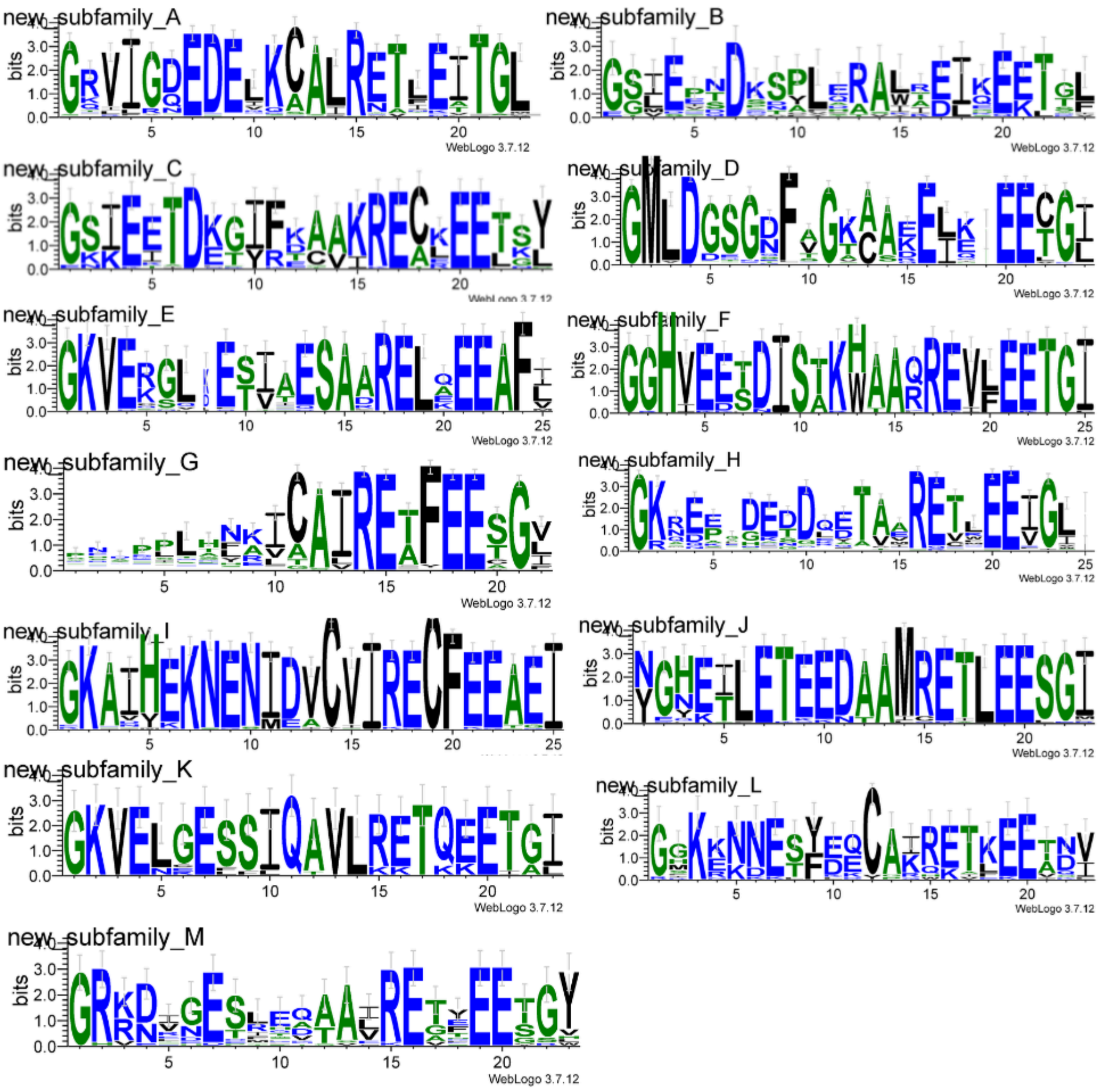

Supplementary Figure S4. NUDIX box motif conservation across all identified NUDIX subfamilies in fungi.

A. NUDIX box motif across all newly identified subfamilies shown as sequence consensus **B.** NUDIX box motif across all newly identified subfamilies obtained with WebLogo 3.7.12.

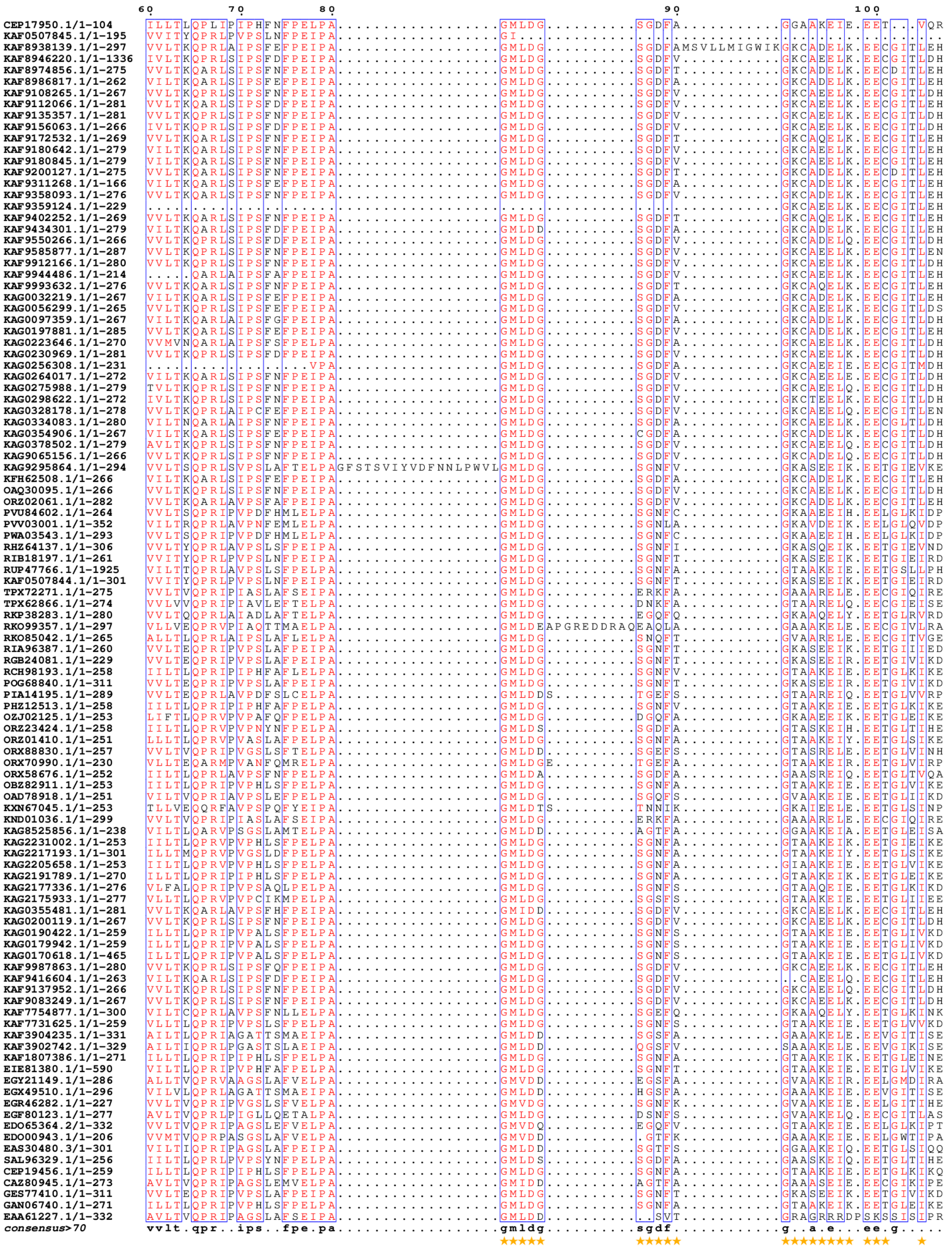

Supplementary Figure S5. Alignment of sequences from new_subfamily_D.

The region of the NUDIX box is marked with orange stars. most of the sequences have conserved motif before the catalytic center: FPExPA.

```
                     1         10                  20        30        40
RHZ45831.1/69-187    .......KPNTPKIWVNKRIN..........PNKEFYKHWQCPGGHVEETDISTKHAAQR
RHZ48206.1/250-371   YSTI....RKQRKYRLVKEQN..........SDKEFYEHWQSPGGHIEESDISAKWAARR
RHZ52810.1/12-137    YTTTVLYDRKTKKIWVSKRTN..........PDKEFYEHWQSPGGHVEESDISTKWAARR
RHZ53956.1/12-127    YVSIILRRPNTPKIWVNKRIN..........PNKEFYKHWQCPGGHVEETDISTKHAAQR
RHZ56541.1/12-137    YVSIILRRPNTPKIWVNKRIN..........PNKEFYKHWQCPGGHVEETNISTKHAAQR
RHZ57731.1/12-137    YVSIILRRPNTPKIWVNKRIN..........PNKEFYKHWQCPGGHVEETDISTKHTAQR
RHZ57732.1/12-137    YVSIILRRPNTPKIWVNKRIN..........PNKEFYKHWQCPGGHVEETDISTKHTAQR
RHZ58156.1/12-137    YVSIILRRPNTPKIWVNKRVN..........PNKEFYKHWQCPGGHVEDTDISAKHAAQR
RHZ58196.1/12-137    YTTTVLHDKKTKKIWVSKRTN..........PNKEFYEHWQSPGGHVEESDISAKWAARR
RHZ62880.1/12-137    YVSIILRRPNTPKIWVNKRIN..........PNKEFYKHWQCPGGHVEKTDISTKHAAQR
RHZ63037.1/12-137    YTTTVLYDKKTKKIWVSKRTN..........PDKEFYEHWQSPGGHIEESDISAKWAARR
RHZ66594.1/610-735   YTTTVLYDKKTKKIWVSKRTN..........PDKEFYEHWQSPGGHVEESDISTKWAARR
RHZ69292.1/12-96     YTTTVLYDKKTKKIWVSKRTN..........PDKEFYEHWQSPGGHVEESDISAKWAARR
RHZ72504.1/12-137    YVSIILRRPNTPKIWVNKRIN..........PNKEFYKHWQCPGGHVEETDISTKHAAQR
RHZ75106.1/12-137    YTTTVLYDKKTKKIWVSKRTN..........PNKEFYEHWQSPGGHIEESDISAKWAARR
RHZ76086.1/737-862   YTTIVLYDRKTKKIWVSKRTN..........PDKEFYEHWQSPGGHVEESDISAKWAARR
RHZ76144.1/12-137    YVSIILRRPNTPKIWVNKRIN..........PNKEFYKHWQCPGGHVEETDISTKHAAQR
RHZ77245.1/12-137    YTTTVLYDRKTKKIWVSKRTN..........PDKEFYEHWQSPGGHVEESDISAKWAARR
RHZ77815.1/12-136    YVSIILRRPNTPKIWVNKRIN..........PNKEFYKHWQCPGGHVEETDISTKHAAQR
RHZ78222.1/12-137    YVSIILRRPNTPKIWVNKRIN..........PNKEFYRHWQCPGGHVEDIDISTRHAAQR
RHZ78629.1/12-137    YVSIILRRPNTPKIWVNKRIN..........PNKEFYKHWQCPGGHVEETDISTKHAAQR
RHZ78630.1/12-129    YVSIILRRPNTPKIWVNKRIN..........PNKEFYKHWQCPGGHVEETDISTKHAAQR
RHZ82225.1/84-160    YTTTVLYDKKTKKIWVSKRTN..........PDKEFYKHWQSPGGHVEESDISTKWAARR
RHZ82943.1/478-602   ...........PKQYKNKRIYIHHITKTKHFKNMKFYKHWQCSGGHVEDTDISAKHAAQR
RHZ83734.1/12-101    YTTTVLYDKKTKKIWVSKRTN..........PDKEFYEHWQSPGGHVEESDISTKWTARR
RHZ84765.1/12-117    YVSIILRRPNTPKIWVNKRIN..........PNKEFYKHWQCPGGHVEETDISTKHAAQR
RHZ84767.1/12-137    YVSIILRRPNTPKIWVNKRIN..........PNKEFYKHWQCPGGHVEETDISTKHAAQR
RHZ85202.1/12-137    YVSIILRRPNTPKIWVNKRIN..........LNKEFHKHWQCPGGHVKDTDISAKHAAQR
RHZ86637.1/82-155    YVSIILRRPNIPKIWVNKRIN..........PNKEFYKHWQCPGGHVEDTNISTKHAALR
RHZ86964.1/12-137    YTTTVLYDKKTKKIWVSKRTN..........PDKEFYEHWQSPGRHVEESDISTKWAARR
RHZ87345.1/12-105    YVSIILRKPNTPKIWVNKRIN..........PNKEFYKHWQCPGGHVEETDISTKHAAQR
RHZ87367.1/12-137    YVSIILRRPNTPKIWVNKRIN..........PNKEFYKHWQCPGGHVEETDISTKHAAQR
RHZ87385.1/12-137    YTTTVLYDKKTKKIWVSKRTN..........PNKEFYEHWQSPGGHIEESDISAKWAARR
RHZ88322.1/12-120    YTTTVLYDKKTKKIWVSKRTN..........PDKEFYEHWQSPGGHVEESDISTKWAAKR
RHZ88644.1/120-213   YTTTVLYDKKTKKIWVSKRTN..........PDKEFYEHWQSPGGHVEESDISTKWAARR
RHZ89132.1/12-137    YTTTVLYDKKTKKIWVSKRTN..........PNKEFYEHWQSPGGHIEESDISAKWAARR
consensus>70         y...il....t.Kiwv.Kr.n..........p#keFy.HWQ.pGgH!ee.#IS.k.aA.R
                                                                  ★★★★★★★★★★★★★★★★

                           50        60        70        80        90        100
RHZ45831.1/69-187    EVLEETGLQPELEELEYMRTNHYFREKEWRIVHCYSFTTRKVPELTEPREMTDWQLKKIK
RHZ48206.1/250-371   EVFEETGIYLKLEELNYWRTQHYYKEDQWRIVHCYKAEIKGIPNLTEPQEMTHWELKKSK
RHZ52810.1/12-137    EVFEETGIYLKLEELNYWRTQHYYKEDQWRIVHCYKAEIKGIPNLTEPQEMTHWELKKSK
RHZ53956.1/12-127    EVLEETGIQPELEELEYMRTNHYFREKEWRIVHCYSLTTKKVPELTEPREMTDWQLKKIK
RHZ56541.1/12-137    EVLEETGIQPELEELEYMRTNHYFREKEWRIVHCYSLTTKKVPELTEPREMTDWQLKKIK
RHZ57731.1/12-137    EVLEETGIQPELEELEYMRTNHYFREKEWRIVHCYSLTTKKVPELTELREMTNWQLKKIK
RHZ57732.1/12-137    EVLEETGIQPELEELEYMRTNHYFREKEWRIVHCYSLTTKKVPELTELREMTNWQLKKIK
RHZ58156.1/12-137    EVLEETGIRSELEELEYTRTNHYFREKEWRIVHCYSLTTKKVPELTEPREMTDWQLKKIK
RHZ58196.1/12-137    EIFEETGIYLKLEELNYWRTRHYYKEDQWRIVHCYKAEIKGIPNLTEPQEMTHWELKKSK
RHZ62880.1/12-137    EVLEETGIQPELEELEYMRTNHYFREKEWRIVHCYSLTTKKVPELTEPREMTDWQLKKIK
RHZ63037.1/12-137    EIFEETGIYLKLEELNYWRTQHYYKEDQWRIVHCYKAEIKGIPNLTEPQEMTHWELKKNK
RHZ66594.1/610-735   EVFEETGIYLKLEKLNYWRTQHYYKEDQWRIVHCYKAEIKGIPNLTEPQEMTHWELKKSK
RHZ69292.1/12-96     EVFEETGIYLKLEELNYWKTQHYYKENQWRIV............................
RHZ72504.1/12-137    EVLEETGIQPELEELEYMRTNHYFREKEWRIVHCYSLTTKKVPELTEPREMTDWQLKKIK
RHZ75106.1/12-137    EVFEETGIYLRLEELNYWRTQHYYKEDQWRIVHCYKAEIKGIPNLTEPQEMTHWELKKNK
RHZ76086.1/737-862   EVLEETGIYLKLEELNYWRTQHYYKEDQWRIVYCYKAEIKGMPNLTEPQEMTHWELKKSK
RHZ76144.1/12-137    EVLEETGIQPELEELEYMRTNHYFREKEWRIVHCYSLTTKKVPELTEPQEMTDWQLKKIK
RHZ77245.1/12-137    EVFEETGIYLRLEELNYWRTQHYYKEDQWRIVHCYKAEIKGMPNLTEPQEMTHWELKKSK
RHZ77815.1/12-136    EVLEETGIQPELEELEYMRTNHYFREKEWRIVHCYSLTTKKVPELTEPREMTDWQLKKIK
RHZ78222.1/12-137    EVLEKTGIQSELEELEYIKTNHYFREKEWRIVHCYSLTTKKVPRLTEPREMTDWQLKKIK
RHZ78629.1/12-137    EVLEETGIQPELEELEYMRTNHYFKEKEWRIIHCYSLTTKKVPELTEPREMTDWQLKKIK
RHZ78630.1/12-129    EVLEETGIQPELEELEYMRTNHYFREKEWRIVH..SIRKEKEEEIKEYQGLFETYQKQEE
RHZ82225.1/84-160    EVFEETGIYLKLEELNYWRTQHYYIRN.................................
RHZ82943.1/478-602   EVLEETGIQSELEEVEYIKTNHYYREKEWRIIHCYSLTTKKVPKLTEPREMTDWQLKKIK
RHZ83734.1/12-101    EVFEETGIYLKLEELNYWRTQHYYKEDQWRIVHCYKAEIK....................
RHZ84765.1/12-117    EVLEETGIQPELEELEYMRTNHYFREKEWX....YKQPCRECQE................
RHZ84767.1/12-137    EVLEETGIQPELEELEYMRTNHYFREKEWRIVHCYSLTTKKVPELTEPREMTDWQLKKIK
RHZ85202.1/12-137    EILEETGIQPELEELEFIRTNYYFREKEWKIVHCYSLTTKKVPKLIEPREITDWQLKKIK
RHZ86637.1/82-155    KVLEETGIQPELEELEYMRTNHYF....................................
RHZ86964.1/12-137    EVFEETGIYLKLEELNYWRTQHYYKEDQWRIVHCYKVEIKGIPNLTEPQEMTHWELKKSK
RHZ87345.1/12-105    EVLEETGIQPELEELEYMRTNHYFREKEWRIVHCYSLTTKKVQN................
RHZ87367.1/12-137    EVLEETGIQPELEELEYMRTNHYFREKEWRIVYCYSLTTKKVPELTEPREMTDWQLKKIK
RHZ87385.1/12-137    EVFEETGIYLRLEELNYWRTQHYYKEDQWRIVHCYKAEIKGIPNLTEPQEMTHWELKKNK
RHZ88322.1/12-120    EVFEETGIYLKLEELNYWRTQHYYKEDQWRIVHCYKAEIKGIPNLTEPQEMTHWELKKR.
RHZ88644.1/120-213   E..............NRPLLQSGNKRN..................TKLNGTTRNDTLGIK
RHZ89132.1/12-137    EVFEETGIYLRLEELNYWRTQHYYKEDQWRIVHCYKAEIKGIPNLTEPQEMTHWELKKNK
consensus>70         ev.eetgi...leel#y.rtnhyf.e.ewrivhcy....k.vpeltep.emt.wqlkk.k
                     ★★★★★★★★
```

Supplementary Figure S6. Alignment of sequences from new_subfamily_F.

The region of the NUDIX box is marked with orange stars. Conserved residues between all proteins are highlighted in red.

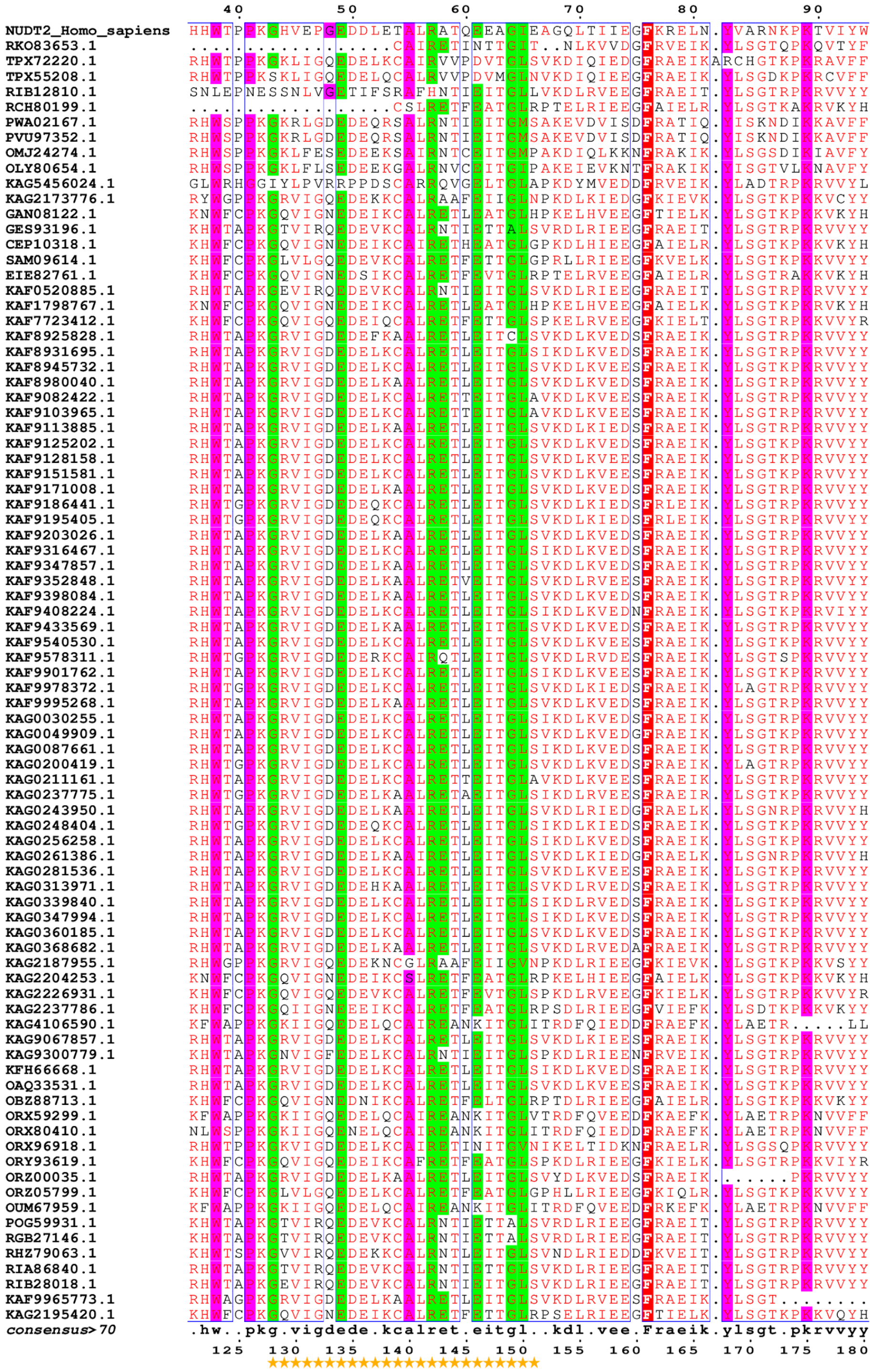

Supplementary Figure S7. Alignment of sequences from new_subfamily_A and human NUDT2 (PDB: 3U53).

Ten residues from the NUDIX box there is phenylalanine typical at this position for NUDT2. Residues in magenta retain conserved residues typical for Ap4Ases. In green are highlighted conserved residues from NUDIX box. The region of the NUDIX box is marked with orange stars.

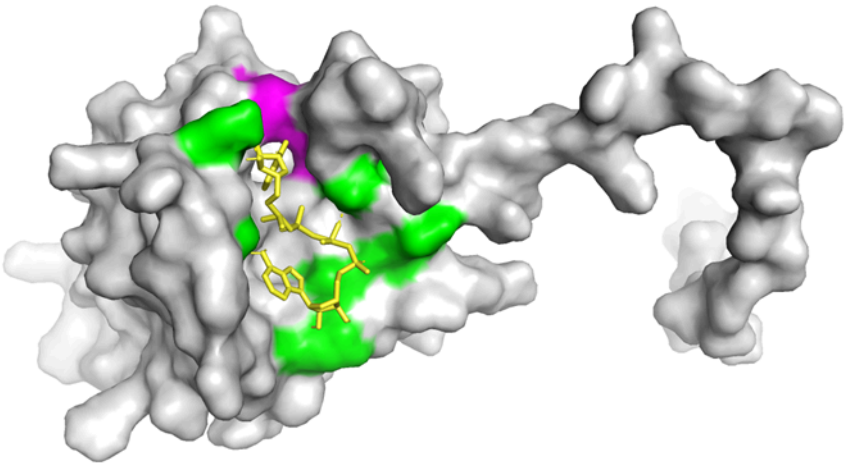

Supplementary Figure S8. Molecular docking of the NUDIX domain with Ap4A.

Representatives of the newly identified subfamily A have high affinity to Ap4A and conserved features of Ap4Aases. Scheme showing the molecular docking of the NUDIX hydrolase domain-containing protein (EIE82761.1) (grey) from *Rhizopus delemar* with Ap4A (yellow) at pH 9.0. Corresponding residues that lie on top of the active site cleft in human NUDT2 are marked in magenta (Tyr461 and Phe507). Other binding residues (Asp409, Lys414, Gln423, Glu437, Glu440 and Lys468) are shown in green.

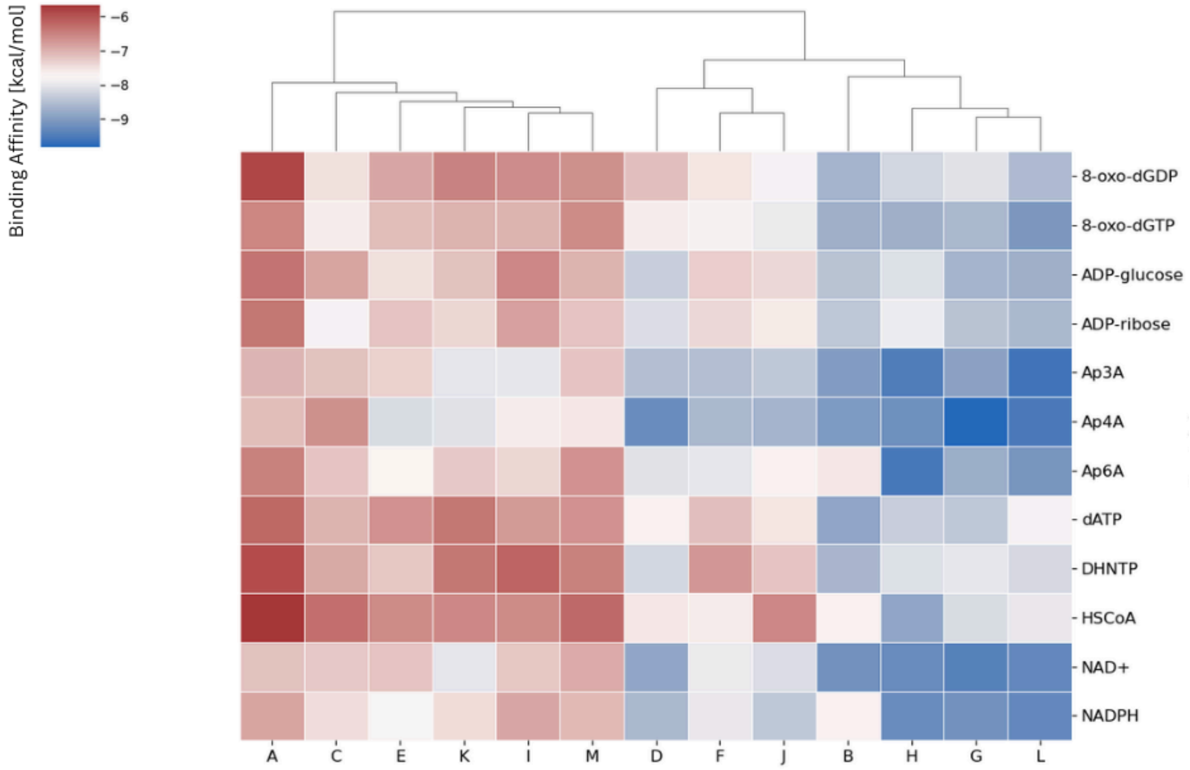

Supplementary Figure S9. Heatmap clustering of binding affinities

Molecular dockings show similarities in the binding affinities of the newly identified NUDIX fungal subfamilies. Molecular docking results for newly identified subfamilies against 12 substrates clustered by similarity between subfamilies. Results represent relative binding energy [kcal/mol].

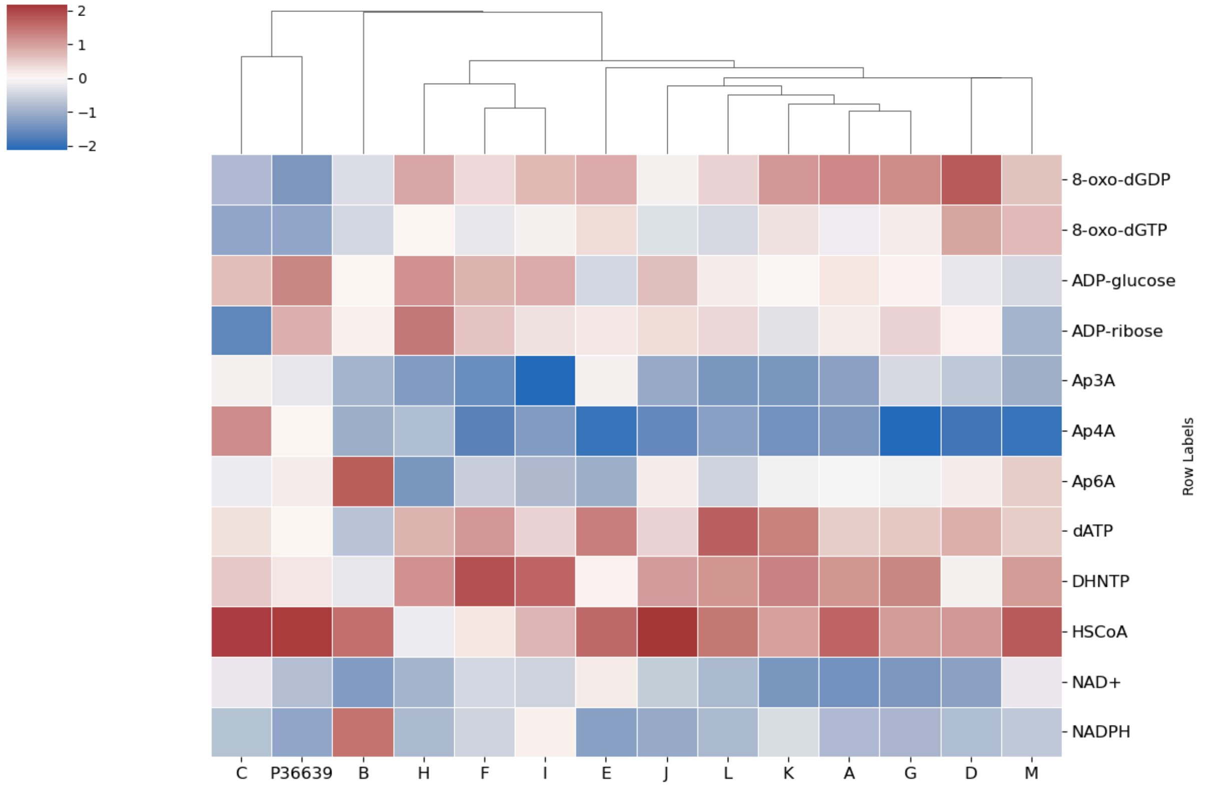

Supplementary Figure S10. Heatmap clustering of binding affinities with control enzyme (NUDT1 from *Homo sapiens* - UniProt: P36639) at pH 8.0

Molecular docking results for newly identified subfamilies against 12 substrates and NUDT1 clustered by similarity between subfamilies. Results represent relative binding energy [kcal/mol] and are normalized by Z-scores with lower values indicating more favorable interactions as previously described.

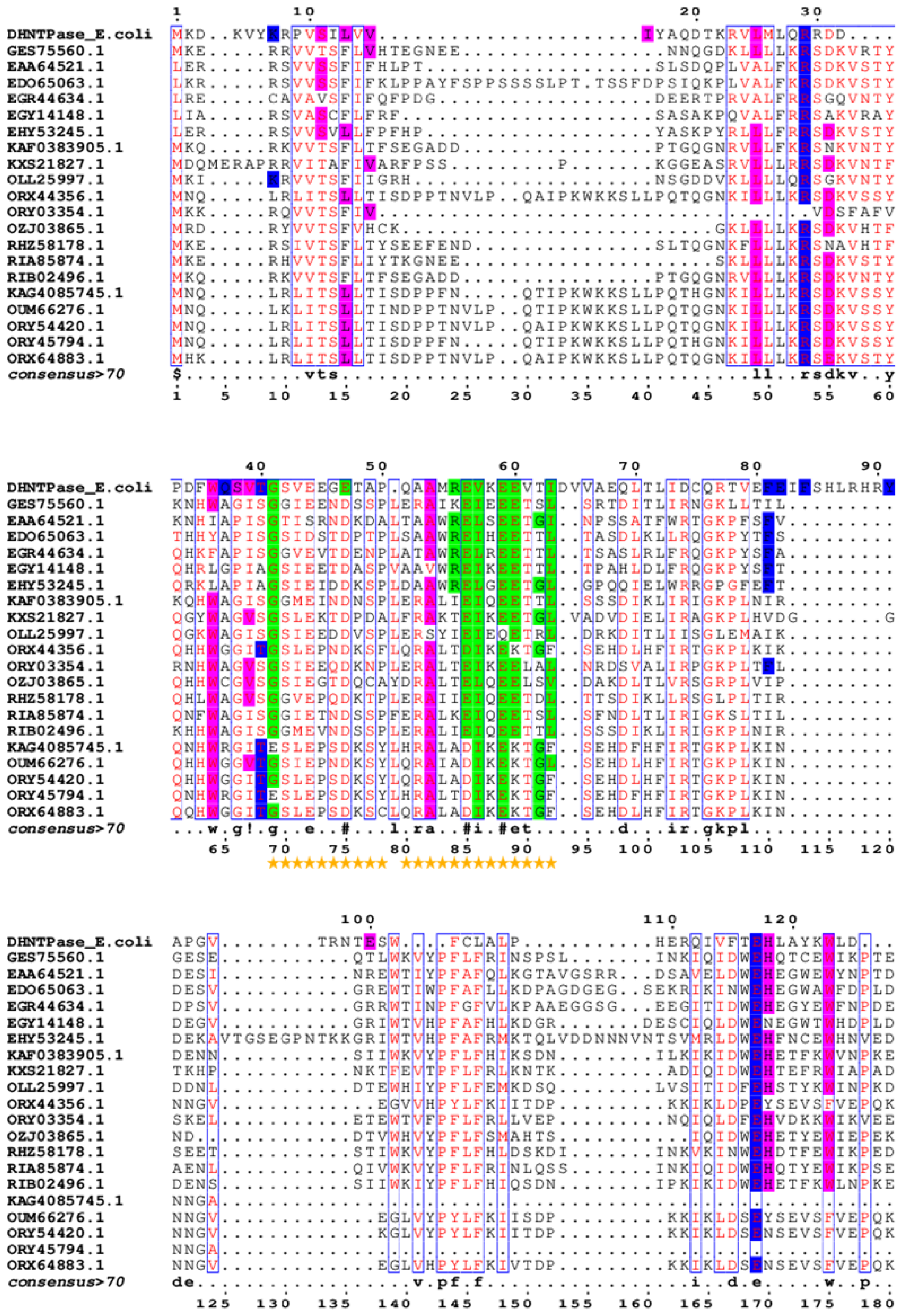

Supplementary Figure S11. Conservation of DHNTPase residues

Typical DHNTPase residues are conserved only within the NUDIX box in the proteins from subfamily B. MSA (Multiple Sequence Alignment)of DHNTPase from E. coli (PDB 2O1C)

and sequences from subfamily_B. The conserved residues from the Nudix signature box are indicated in green, and the ones implicated in recognition or catalytic mechanisms are highlighted in blue. Other conserved residues are depicted in magenta. The region of the Nudix box is marked with orange stars. This Supplementary Figure Swas made with the programs MAFFT v7.407 (W. Li et al., 2001; Rozewicki et al., 2019) and ESPript (Robert & Gouet, 2014). The conserved residues in DHNTPase from E. coli are based on (Gabelli et al., 2007).

Supplementary characterization of subfamilies

The cluster of **subfamily_A** includes representatives from the fungal phyla Chytridiomycota, Microsporidia, Mucoromycota, Olpidiomycota, and Zoopagomycota. Apart from the canonical NUDIX (PF00293) domain these proteins also contain two copies of the zinc finger C-x8-C-x5-C-x3-H domain (PF00642) and, in most cases, also the translation initiation factor eIF4-gamma/eIF5/eIF2-epsilon domain (PF02020). Almost all sequences from this subfamily have conserved residues typical for Ap4Ases such as NUDT2 from *Homo sapiens* (PDB: 3U53): Trp38, Pro41, Gly43, Lys89, and Tyr82 (Ge et al., 2013; Srouji et al., 2017). Moreover, ten residues downstream from the NUDIX box, these enzymes have a conserved phenylalanine which is also present in the human NUDT2 protein (see Supplementary Figures section 1). Molecular docking results of representatives of this subfamily showed high activity towards NAD+, Ap4A, and Ap3A. As proteins from this subfamily do not contain characteristics of NADH pyrophosphatases or diphosphatases (NADD) such as the SQPWPFPXS motif, it is unlikely that they hydrolyze NAD+. This concludes that NUDT2 is known not to have de-NAD-ing activity (Sharma et al., 2020). On the other hand, as can be observed in Figure 6, the two residues needed for docking of Ap4A, Tyr461 and Phe507, lie on top of the active site, as they do in human NUDT2. The residues from within the NUDIX motif also interact with Ap4A (Figure 6). These findings strongly suggest that the substrate of proteins of **subfamily_A** is Ap4A and, as human NUDT2, their role may be to maintain the intracellular level of Ap4A ensuring the viability of cells in adverse environments (Zegarra et al., 2023).

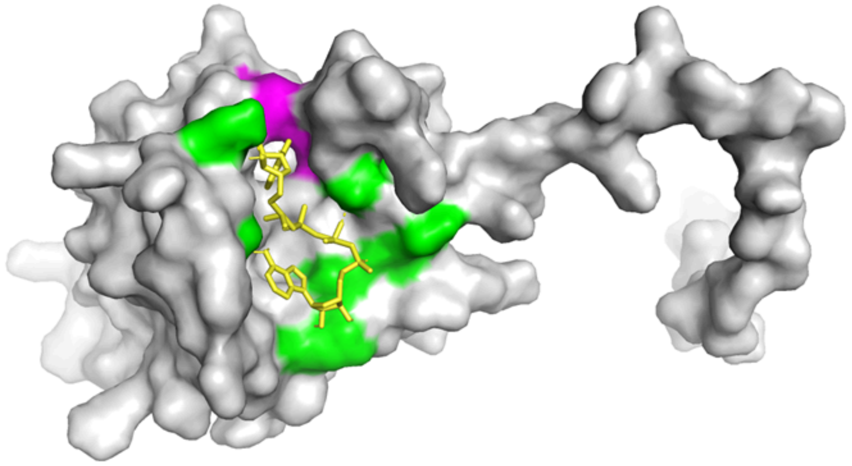

Supplementary Figure S8. Representatives of the newly identified subfamily A have high affinity to Ap4A and conserved features of Ap4Aases. Scheme showing the molecular docking of the NUDIX hydrolase domain-containing protein (EIE82761.1)(grey) from *Rhizopus delemar* with Ap4A (yellow) at pH 9.0. Corresponding residues that lie on top of the active site cleft in human NUDT2 are marked in magenta (Tyr461 and Phe507). Other binding residues (Asp409, Lys414, Gln423, Glu437, Glu440 and Lys468) are shown in green.

Sequences from the **subfamily_B** have an elongated NUDIX box motif by one residue, and a recurrent coexisting domain, the translation initiation factor 2B subunit (PF1008) essential for eukaryotic translation initiation. In fungi, we identified members of this subfamily in the phyla Ascomycota, Chytridiomycota, Mucoromycota, and Zoopagomycota, and there are also some representatives in bacteria and archaea. The sequences from this cluster map to the DHNTPase-like enzymes (cd04664) according to the NCBI CDD database, however, they do not retain sequence motifs characteristics for such hydrolases. Furthermore, we compared the conserved residues typical for DHNTPases with the sequences of this subfamily (Supplementary Figures Section 7) and found that the residues and the characteristic alanine for DHNTPase within the NUDIX box are quite well-conserved. However, outside of the NUDIX box, the sequence is quite divergent. Moreover, typical dihydroneopterin triphosphate pyrophosphatases stack the substrate's ring into a hydrophobic pocket consisting of Phe81 and Phe84 (Gabelli et al., 2007), and these residues were not conserved in this subfamily. Finally, molecular docking of representatives of the subfamily confirmed that DHNTP is not likely a preferred substrate. Since the neighboring domains are essential for translation, we rather think that proteins from this subfamily maintain the housekeeping nature of NUDIX hydrolases.

Sequences from **subfamily_C** are found only in species of the order *Diversisporales.* These enzymes have an elongated NUDIX box by the insertion of an aspartate (D): $\mathbf{G}x_5Dx_8\mathbf{RE}x_2\mathbf{EE}x_3$ (conserved residues from the NUDIX box are marked in the bold, see Supplementary Figures section 2). 62.5% of these hydrolases have a deoxynucleoside kinase domain (PF01712) in addition to the NUDIX motif. Their predicted subcellular localization is the nucleus suggesting that they may hydrolyze nucleotides. Proteins from **subfamily_D** have sequence similarity to a *provisional plant NUDIX subfamily* in the CDD database (PLN03143). This may be because a small fraction of the representatives of this subfamily are from Protozoa and plants, although most of the proteins in the subfamily are fungal. Most of the proteins of **subfamily_D** belong to early-diverging fungal phyl,a and they have a strongly conserved pattern in their NUDIX box: $\mathbf{GMLD}x_4\mathbf{F}x_6\mathbf{E}x_2\mathbf{EE}x\mathbf{GU}$ (conserved residues marked in bold).

Proteins of **subfamily_E** are related to the human NUDIX enzyme NUDT1, although all members of the cluster are fungal proteins. Their position in the phylogenetic tree suggests that they may have originated as a result of a post-duplication diversification of NUDT1 paralogs. However, these enzymes do not retain all characteristics that are distinctive of NUDT1. For example, the substrate affinity calculated through molecular docking is low for substrates that are typical of NUDT1 such as 8-oxo-dGTP, 8-oxo-dGDP, or dATP. This suggests that members of this subfamily probably operate distinctly from NUDT1.

Subfamily_F groups NUDIX hydrolases only from a single species from the phylum Glomeromycota, *Diversispora epigaea,* that have the following NUDIX box signature motif: **GGHVEE**x**DIS**x**K**x**AA**x**REU**x**EE**x**GU** (in bold black are residues conserved from NUDIX box motif, in bold green residues conserved across most of the sequences in this family). Furthermore, this motif is preceded by two conserved lysines situated four residues apart and by a conserved histidine-tryptophan-glycine triplet of three residues from the NUDIX box (see Supplementary Figures section 4). Molecular docking showed a relatively high affinity for ApnA substrates, but the binding was not through the residues from within the NUDIX box making it unlikely that this subfamily hydrolases ApnA. All proteins are predicted to be localized in the nucleus and therefore they could be hydrolyzing nucleotides.

Both **subfamily_G** and **subfamily_H** were associated with human NUDT7 CoAse (cd03426) by NCBI CDD and Foldseek. Subfamily_G has representatives from bacteria, and a few insects apart from the fungal proteins, while subfamily_H is almost exclusively fungal with a few members from bacteria.However, molecular docking predicted that Coenzyme A is not the preferred substrate of the enzymes in these subfamilies, showing functional divergence from NUDT7.

Subfamilies I and **J** only include sequences from a single species from the phylum Glomeromycotina, *G. rosea*. According to NCBI CDD search, these enzymes have either NUDT1MTH1 (cd03427) or NUDT15MTH2 (cd04678) characteristics, which correspond to human NUDIX enzymes NUDT1 and NUDT15, respectively. In humans, these hydrolases are known to oxidize deoxyribonucleoside di- and triphosphates to preserve genomic integrity (Carter et al., 2015; Hashiguchi et al., 2018). Both subfamily genes are often found close to kinase-like domain-containing genes. However, the predicted structures of the fungal proteins

from these subfamilies lack the typical NUDT15 recognition pocket and the Arg139 residue that is important for thiopurine metabolism. Furthermore, these enzymes also do not have the conserved residues characteristic of human NUDT1 and molecular docking did not indicate high affinity towards the canonical NUDT1 or NUDT15 substrates. Even though there are structural differences between proteins from these subfamilies and NUDT1 and NUDT15, the highly conserved NUDIX box may indicate these proteins are engaged in housekeeping functions.

subfamily_K groups fungal proteins of order *Glomerales,* but there are also representatives in bacteria. In addition to the canonical NUDIX domain, 57% of sequences have a retroviral aspartyl protease domain (PF08284) and 21% have a zinc knuckle domain (PF00098). The viral nature of the coexisting domains suggests that the domain architecture of this subfamily may be the consequence of the fusion of a NUDIX protein with a retrotransposon. Species of the *Rhizophagus* genus are known to have experienced massive transposon activity, causing that half of the genomic sequence to be some kind of repeat. The subcellular localization of the fungal sequences in this subfamily is predicted to be mostly the nucleus, perhaps due to the fusion with a retrotransposon. In contrast, human NUDT15, (which was annotated by NCBI CDD as a possible homolog of the proteins of this subfamily) is known to reside in the cytosol. Despite its possible association with retrotransposons, molecular docking showed that binding with 8-oxo-dGTP occurs through the residues that correspond to the binding pocket in NUDT15. In consequence, the members of this subfamily may be involved in thiopurine metabolism.

As for subfamily_C, **subfamily_L** only groups sequences from the phylum Glomeromycotina, particularly the *Diversisporales* order. Most NUDIX hydrolases in this cluster are predicted to be in the nucleus.

Contrary to most newly identified subfamilies, **subfamily_M** includes proteins mainly from Ascomycota and Basidiomycota and a small subset of proteins from Bilateria and bacteria. Similar to the characterized Ap4Ases identified in the yeast species *Schizosaccharomyces pombe* and *Saccharomyces cerevisiae*, proteins of this subfamily lack the conserved tyrosine situated 16–18 amino acids after the NUDIX box (Dunn et al., 1999). The docking results ranked Ap3A and Ap4A as substrates with the lowest changes in free energy during binding, making them possible ligands. Therefore, enzymes from **subfamily_M** might be Ap4ases involved in the diadenosine polyphosphate catabolic

process, similar to the function of previously characterized yeast enzymes (Dunn et al., 1999). Based on their phylogenetic position, the members of **subfamily_M** are related to the human NUDT2 enzyme. According to the tree, proteins of **subfamily_M** diverged from the same branch that gave rise to different human hydrolases from the canonical NUDIX family (NUDT2, NUDT6, and NUDT20), and also to 39S mitochondrial ribosomal proteins L46 that belong to the MRP-L46 family (PF11788), another member of the NUDIX superfamily.
